# *Bacillus cereus* enterotoxins change epithelial barrier function and ion transport in a Caco-2/HT29-MTX co-culture model, provoking inflammation, apoptosis and membrane damage

**DOI:** 10.1080/21505594.2026.2725391

**Published:** 2026-08-27

**Authors:** Manar Albahri, Bettina Seeger, Andreas Beineke, Tuba Esatbeyoglu, Madeleine Plötz, Nadja Jessberger

**Affiliations:** a Institute of Food Quality and Food Safety, University of Veterinary Medicine Hannover, Hanover, Germany; b Department of Pharmacology, Toxicology, and Pharmacy, University of Veterinary Medicine Hannover, Hanover, Germany; c Centre for Systems Neuroscience, University of Veterinary Medicine Hannover, Hanover, Germany; d Department of Pathology, University of Veterinary Medicine Hannover, Hanover, Germany; e Department of Molecular Food Chemistry and Food Development, Institute of Food and One Health, Gottfried Wilhelm Leibniz University Hannover, Hanover, Germany

**Keywords:** Host intestine, toxico-infection, RNA sequencing, cellular pathways, cell death, cell recovery

## Abstract

Diarrheal disease caused by *Bacillus cereus* is driven by pore-forming enterotoxins, with hemolysin BL (Hbl) and non-hemolytic enterotoxin (Nhe) as major virulence factors. Toxin structure and mode of action are well described, while especially human intestinal epithelial responses remain incompletely characterized. With the co-culture system of Caco-2 and mucus-producing HT29-MTX cells (9:1), this study describes a suitable model for investigating enterotoxin effects. Cell line-, dose- and enterotoxin-dependent changes in cell viability were detected. Undifferentiated Caco-2 generally showed higher susceptibility than HT29-MTX cells. Twenty-four-hour co-cultures were more susceptible than 28-day co-cultures but displayed partial recovery after toxin removal. Enterotoxin exposure caused a time-dependent decline in transepithelial electrical resistance, consistent with barrier dysfunction. In Ussing chamber experiments, enterotoxin-containing supernatants, especially Nhe+Hbl, increased short-circuit currents, indicating altered electrogenic ion transport consistent with pro-secretory phenotypes relevant to diarrhea. Transcriptome analyses demonstrated a pronounced time- and dose-dependent response toward Nhe+Hbl. Two-hour sublethal enterotoxin exposure elicited few transcriptional changes, whereas lethal enterotoxin exposure rapidly suppressed proliferation- and cell-cycle-associated programs. Pathway analyses consistently highlighted TNFα/NF-κB-driven inflammation, MAPK-associated stress signaling, and p53/apoptosis pathways; metabolic remodeling and senescence-/autophagy-related processes became more apparent at longer exposure (8 h) and/or higher toxin levels. Dose-dependent caspase-3/7 activation, LDH release, and minimal caspase-1 activation were also consistent with early inflammatory response, apoptotic cell death and membrane damage. Collectively, this study integrates barrier function, ion transport, cell viability, and transcriptomics to define host intestine epithelial response programs triggered by *B. cereus* enterotoxins and to connect toxin exposure to barrier dysfunction and diarrheal pathophysiology.

## Introduction

*Bacillus cereus* is a Gram-positive, spore-forming bacterium that is ubiquitous in soil, dust, and thus, a wide variety of foods. Due to its ability to form heat- and desiccation-resistant spores, it can persist along the entire food chain and therefore represents an important cause of foodborne illness [1]. Two distinct gastrointestinal syndromes are associated with *B. cereus*: the emetic type, caused by the heat-stable, cyclic dodecadepsipeptide cereulide, and the diarrheal type, which is mediated by proteinaceous enterotoxins produced in the small intestine after ingestion of contaminated food [2]. The diarrheal syndrome has been primarily attributed to three pore-forming toxins: hemolysin BL (Hbl) [3,4], non-hemolytic enterotoxin (Nhe) [5], and cytotoxin K (CytK) [6]. The highly toxic variant of this β-barrel pore-forming toxin, CytK-1, has been found only in a few strains that have been classified as separate species, *Bacillus cytotoxicus* [7].

Both Hbl and Nhe are tripartite toxins requiring the coordinated action of their protein components for full activity. These toxins act by sequential binding to the plasma membrane, oligomerization, and pore formation, resulting in osmotic imbalance, cell swelling, and lysis [8,9]. Hbl is built from one binding (B) and two lytic (L1, L2) subunits, whereas Nhe is composed of the subunits A, B, and C. The optimum concentration ratio for rapid Hbl pore formation is L2:L1:B = 1:1:10 or 10:1:10, in contrast to Nhe with A:B:C = 10:10:1 [8,10]. The respective subunits assemble in a linear and specific order to form the pore, which is B-L1-L2 for Hbl and C-B-A for Nhe. Here, it is important to note that individual subunits form complexes even before cell contact. For example, complex formation with L1 enhances the binding of Hbl B to the cell membrane [8], and NheC is found almost exclusively in complexes with NheB [11]. In addition, NheB and C already form small transmembrane channels of approx. 2 nm diameter, which were designated as pre-pores [12]. The full Nhe pores were characterized as large, >2.8 nm, stable, and cation-selective [13,14]. Hbl pores are also cation-selective, but small, approx. 1.2 nm, unstable and of limited lifespan [15].

At the host–toxin interaction level, a previous study identified LPS-induced TNFα factor (LITAF) as the main receptor for Hbl as well as cell death-inducing p53 target 1 (CDIP1) as an alternative receptor [16]. Although the receptor for Nhe still needs to be identified, evidence exists for attachment of Nhe-containing extracellular vesicles to intestinal epithelial cells via cholesterol-rich domains and dynamin-mediated endocytosis. Further, these vesicles triggered inflammatory response in human monocytes [17]. An earlier study showed that Nhe pore formation leads to influx of extracellular Ca^2+^ and triggers apoptotic cell death through activation of apoptosis signal-regulating kinase 1 (ASK1) and p38 MAPK-mediated, caspase-8-dependent pathways [18]. Additionally, Hbl and Nhe were reported to activate inflammasome-mediated mortality via sensor protein NLRP3 (NOD-, LRR- and pyrin domain-containing protein 3), ultimately leading to pyroptosis, an inflammatory form of cell death [19–21].

While the structural and functional properties of Hbl and Nhe are already well characterized, there is still a lack of information regarding the onset of the diarrheal syndrome after enterotoxin production by *B. cereus* in the intestine. Due to their high sensitivity, Vero (African green monkey kidney epithelial) cells were used to routinely determine the cytotoxicity of *B. cereus* isolates or to understand initial signaling pathways [18]. Other studies assessed function and effect of Hbl and Nhe on macrophages [9,20,21] or Chinese hamster ovary cells [9]. Caco-2 cells were also used as a first, simple model representing the human intestinal epithelium [17,22]. Caco-2 cells, derived from a human colorectal adenocarcinoma, represent a well-characterized cell culture system that displays enterocyte-like morphology and functionality, comparable to the small intestine, when differentiated by using certain culture conditions [23]. Nevertheless, traditional Caco-2 monocultures lack critical features such as mucus production [24]. Mucus-producing goblet cells are the second most abundant intestinal cell type [25]. The mucus layer plays a dual role by acting as a physical barrier and by modulating the diffusion and interaction of toxins with epithelial cells [26].

To better mimic the intestinal microenvironment, co-culture systems combining Caco-2 with mucus-producing HT29-MTX cells were developed. A Caco-2:HT29-MTX ratio of 9:1 proved to be best suited in this model, which has been increasingly used to investigate drug absorption, microbial adhesion, and toxin effects [24,27–30]. Differentiation for at least 21 d ensures full polarization of the enterocyte-like cells and maximal mucus production by the goblet-like cells, thereby establishing a physiologically relevant intestinal epithelial model [28]. The present study utilizes this system to elucidate the effects of the tripartite *B. cereus* enterotoxins on the intestinal epithelium, including binding to the cell surface, membrane integrity, ion transport and cell viability. This involves not only comparisons between eukaryotic cell types, but also between Nhe- and Hbl-containing *B. cereus* culture supernatants. Furthermore, this study aims to understand cellular responses to enterotoxin exposure on a transcriptional level, particularly the balance between induced cell death and repair mechanisms, as well as the induction of inflammatory and apoptotic signaling pathways.

## Materials and methods

### Bacterial strains, culture conditions and toxin production

Three *B. cereus* strains were used in this study: F837/76 (DSM 4222, acquired from DSMZ, German Collection of Microorganisms and Cell Cultures GmbH, Braunschweig, Germany), which produces comparably high amounts of both Nhe and Hbl [3,31], NVH 0075–95, which produces comparably high amounts of Nhe only [5,31], and the mutant strain F837/76 Δ*nheABC*, producing Hbl, but not Nhe [32]. For the preparation of toxin-rich, cell-free supernatants, bacteria were cultivated for 6 h in casein-glucose-yeast (CGY) medium supplemented with 1% glucose and processed as previously described [33]. Enterotoxins present in the supernatants were subsequently detected using sandwich enzyme immuno assays, according to [32] and [34]. Supernatants were applied in dilutions in cell culture medium as indicated in the individual experiments. Recombinant Hbl (rHbl) proteins L2, L1 and B were overexpressed in *Escherichia coli* BL21 (DE3) and purified via affinity chromatography as described [32]. The concentration of the rHbl components was determined by SDS-PAGE [32] and subsequent Coomassie blue staining. Band intensities were analyzed using a GelDoc™ EZ Imager in combination with Image Lab software (Bio-Rad, Feldkirchen, Germany). For all experiments, rHbl components (L2:L1:B) were mixed at 1:1:10 ratio and applied in concentrations as indicated in the individual experiments.

### Cell lines and culture conditions

Caco-2 and HT29-MTX cells (passages 16–25) were maintained in 80 cm^2^ culture flasks under standard conditions (37°C, 5% CO_2,_ ~100% humidity) in Dulbecco’s Modified Eagle Medium (DMEM) supplemented with 10% fetal bovine serum (FBS), 1% glutamine, 1% non-essential amino acids, and 1% penicillin/streptomycin (all purchased from PAN-Biotech, Aidenbach, Germany). Cells were passaged twice weekly at ~80–90% confluence. For co-culture experiments, Caco-2 and HT29-MTX cells were seeded at a 9:1 ratio and cultivated under the same conditions. For differentiation, the co-culture was cultivated for 21–28 d, and the culture medium was exchanged every 2–3 d.

### Transepithelial electrical resistance measurements

To assess the barrier integrity of the cell monolayer (particularly tight junction/paracellular integrity [35,36]), transepithelial electrical resistance (TEER) was measured using an epithelial voltohmmeter (EVOM^2^; WPI, Friedberg, Germany). For this, the Caco-2/HT29-MTX co-culture was seeded on transwells (12-well, Greiner Bio-One GmbH, Frickenhausen, Germany) at 1 × 10^5^ cells/transwell and incubated for 21 d. Measurements were conducted at each medium exchange according to the manufacturer’s instructions, as well as 20–180 min after toxin exposure. To investigate barrier recovery after toxin-induced damage, the toxin-containing medium was removed and replaced by fresh, toxin-free DMEM. Cells were subsequently incubated at 37°C, 5% CO_2_, ~100% humidity for 21 days and TEER was measured every second day using the EVOM^2^ voltohmmeter. TEER values were expressed in Ω·cm^2^ by multiplying the raw data with the effective membrane surface area. Stable values ranged between 450 and 550 Ω·cm^2^.

### WST-1 bioassays

To assess the cytotoxic activity of enterotoxin-containing *B. cereus* supernatants and rHbl, WST-1 bioassays were performed as previously described [33]. Briefly, serial dilutions of the toxin-containing samples were prepared in 96-well plates (TPP Techno Plastic Products, Trasadingen, Switzerland). Subsequently, Caco-2, HT29-MTX, and Caco-2/HT29-MTX co-cultures were seeded at 2 × 10^4^ cells/well and incubated for 22 h at 37°C and 5% CO_2_. Additionally, the co-culture was incubated for 28 d in the 96-well plate before toxin application. Following addition of WST-1 reagent (Merck, Darmstadt, Germany), absorbance was measured at 450 nm (Infinite® M200, Tecan, Männedorf, Switzerland). Dose-response curves and 50% inhibitory concentrations (IC_50_) were calculated using GraphPad Prism version 10.0.0 for Windows, GraphPad Software, Boston, Massachusetts USA, www.graphpad.com. Cytotoxicity titers are defined as the reciprocal dilution causing a 50% reduction in cell viability compared to untreated cells.

### Flow cytometry

As the examination procedure has already been well-established on Vero cells [8,15], the binding component of Hbl, in this case rHbl B, was selected as an example to investigate putative differences in binding to Caco-2, HT29-MTX and Caco-2/HT29-MTX cells. Binding was tested as previously described [8,32]. Briefly, rHbl B was adjusted to 1.5 pmol/µl, diluted 1:20 in 500 µl extracellular (EC) buffer (140 mM NaCl, 15 mM HEPES, 1 mM MgCl_2_, 1 mM CaCl_2_, 10 mM glucose, pH 7.2), and incubated with 1 × 10^6^ cells in 500 µl EC buffer (final dilution 1:40) at 37°C and moderate agitation for 1 h. After washing in 1% BSA-PBS, cells were incubated with 10 µg/mL monoclonal antibody (mAb) 1G8 [32] for 1 h at room temperature and washed again. Cells were further incubated with 2 µg/mL Alexa Fluor® 488-conjugated goat anti-mouse antibody (Thermo Fisher Scientific GmbH, Dreieich, Germany) for 1 h at 4°C. After final washing, 2 × 10^4^ cells per sample were analyzed by flow cytometry (MACSQuant Analyzer 10; Miltenyi Biotec GmbH, Germany). Negative controls included cells without rHbl B and/or mAb 1G8. Data was further analyzed using FlowJo™ v10.8 Software (BD Life Sciences, Heidelberg, Germany).

### Ussing chamber

To assess toxin-induced changes in epithelial ion transport under controlled electrophysiological conditions, short-circuit current (Isc) measurements were performed on Caco-2/HT29-MTX co-cultures using an Ussing chamber setup, as previously described [28]. Co-cultured cells were grown on Snapwells® (Corning, Kaiserslautern, Germany; diameter 12 mm, pore size 0.4 µm). Cells were seeded at 1 × 10^5^ cells/insert and incubated for 21 d at 37°C, 5% CO_2_. After that, they were mounted in Ussing chambers, representing the mucosal and serosal sides of the humane intestine. Chambers were connected to a computer-controlled voltage/current clamp (Advantech, Hilden, Germany), and each compartment was filled with 4 mL mucosal or serosal buffer solution as described [28]. Chambers were maintained at 37°C and carbogen (95% O_2_/5% CO_2_) was continuously supplied to the buffer to maintain tissue oxygenation, stabilize the pH of the bicarbonate-buffered solution, and ensure adequate mixing during measurements. Following a 15-min equilibration period under short-circuit conditions, enterotoxin-containing *B. cereus* supernatants, diluted 1:40 in buffer, were injected to the apical side of the cell monolayer. Transepithelial current (Isc; indicates electrogenic ion transport) and resistance (Rt; indicates electrical barrier integrity similar to TEER measurements) [37] were subsequently measured over 2.5 h. As negative controls, cells were incubated with either bacterial culture medium (CGY) or rHbl elution buffer. Changes in short-circuit current (ΔIsc) were calculated as the difference between the peak Isc observed after addition of each toxin or medium/buffer and the arithmetic mean of the 10 Isc readings recorded immediately before application. Rt before treatment was calculated as mean of 10 values before treatment with either toxin or medium/buffer. Rt after treatment was defined as the minimal reached Rt after addition of toxins or medium/buffer.

### Microscopy and live cell imaging

Cell proliferation in culture flasks or plates was routinely checked under a light microscope (Eclipse Ti-S Inverted Microscope, Nikon, Düsseldorf, Germany). This was also used to monitor toxin-exposed samples.

To localize rHbl B on the Caco-2 and HT29-MTX cell surfaces, electron microscopy with immunogold labeling was performed as described previously [38,39]. For this, 1 × 10^6^ cells were incubated with rHbl B (1.5 pmol/µl, final dilution 1:60), washed and incubated with mAb 1G8 as described for flow cytometry. After the second washing step, goat anti-mouse IgG–gold conjugate (6 nm, Aurion Immuno Gold Reagents & Accessories, Wageningen, The Netherlands) was added to the cells in 1:50 dilution in 1% BSA-PBS. After 1 h incubation at room temperature and moderate agitation, samples were washed three times, resuspended in 500 µl 1% BSA-PBS, and fixed by adding 500 µl 4% paraformaldehyde and 0.25% glutaraldehyde in cacodylate buffer. Negative controls included cells incubated with both antibodies, but without rHbl B. Cells were further fixated with 2.5% glutaraldehyde and incubated overnight at 4°C. Post-fixation was performed in 1% aqueous osmium tetroxide and after five washes in cacodylate buffer (five min each) samples were dehydrated through a series of graded alcohols and embedded in Epon 812 medium. Ultra-thin sections were cut with a diamond knife (Diatome, Quakertown, USA) on a microtome (Ultracut Reichert-Jung, Leica Microsystems, Germany), transferred to copper grids and stained with uranyl citrate for 15 min. After eight washing steps, samples were incubated with lead citrate for seven min and examined with a transmission electron microscope (Leo 906, Carl Zeiss Microscopy Deutschland GmbH, Oberkochen, Germany).

To visualize cell death and recovery, Caco-2/HT29-MTX co-cultures (5 × 10^5^ cells/well) were seeded in 6-well plates (TPP Techno Plastic Products, Trasadingen, Switzerland) and incubated for 24 h. After that, they were exposed to lethal (IC_90_; 1:40) and sublethal (IC_10_; 1:400 for NVH 0075–95 (Nhe) and F837/76 Δ*nheABC* (Hbl); 1:600 for F837/76 (Nhe+Hbl)) dilutions of *B. cereus* culture supernatants. Dilutions were selected based on viability assay results (WST-1 bioassays). Cells were incubated at 37°C, 5% CO_2_, ~100% humidity for another 24 h before removal of the toxin-containing cell culture medium and addition of fresh medium. They were monitored for another 48 h using CytoSMART™ 2 live cell imaging camera (Lonza, Oss, The Netherlands). Images were taken every 2 min during toxin exposure and every 10 min after toxin removal. From these, videos were created using Microsoft Movie Maker – Video Editor with 0.3 s time between images.

### RNA isolation, mRNA sequencing and data analysis

Caco-2/HT29-MTX co-cultures were grown in 6-well plates (TPP Techno Plastic Products, Trasadingen, Switzerland) for 28 d. After that, the medium was removed and replaced with 3 mL medium containing either sublethal (IC_10_; 1:325) or lethal (IC_90_; 1:35) doses of *B. cereus* F837/76 (Nhe+Hbl) culture supernatant. Dilutions were selected based on viability assay results (WST-1 bioassays). Incubation period at 37°C, 5% CO_2_, ~100% humidity was 2 and 8 h, respectively. All experiments were performed in triplicate. Cells were then mechanically scraped from the well bottoms, and the wells were rinsed twice with 2 mL ice cold PBS. The 7 mL samples obtained were centrifuged for 10 min at 4°C and 3,000 g. Cell pellets were resuspended in 1 mL ice cold PBS, centrifuged again for 10 min at 4°C and 16,000 g and, after removal of the supernatant, stored at −80°C until further usage.

Cell pellets were thawed on ice and resuspended in 500 µl Nucleozol (Macherey-Nagel, Düren, Germany). After 5 min incubation at room temperature, 100 µl chloroform were added. Samples were vortexed for 1 min and subsequently centrifuged for 10 min at 4°C and 20,000 g. The clear supernatant was transferred to a new reaction tube containing an equal amount of 70% EtOH. From there, total RNA preparation and on-column DNase digestion were performed proceeding with step 5 of the Nucleospin RNA II kit (Macherey-Nagel, Düren, Germany) according to the instructions of the manufacturer. RNA concentration and purity were determined using a Nanodrop One spectrophotometer (Thermo Fisher Scientific GmbH, Dreieich, Germany). RNA quality was additionally tested via agarose gel electrophoresis. Contamination with residual DNA was ruled out by PCR against 18S (fw: GCCGGTACAGTGAAACTGC, rev: GTCCGGGCCGGGTGAG, 1210 bp fragment) and ITS1 (fw: GAACTTGACTATCTAGAGGAAG, rev: CAGCTAGCTGCGTTCTTCATC, 1204 bp fragment). RNA sequencing and initial data analyses were performed by Azenta Life Sciences (GENEWIZ Germany GmbH, Leipzig, Germany) using Illumina paired-end sequencing (Illumina NovaSeq X Plus, 2 × 150 bp). Library preparation workflow included rRNA depletion, RNA fragmentation and random priming, first- and second‑strand cDNA synthesis, end repair, 5’ phosphorylation and dA-tailing, as well as adaptor ligation, PCR enrichment and sequencing. Bioinformatics analysis workflow included evaluation of sequence quality, trimming of reads, mapping of reads to the *Homo sapiens* GRCh38 reference genome, generation of hit counts for genes/exons, comparison of exon hit counts as well as gene hit counts, and analysis of gene ontology for the latter. RNA sequencing data were deposited in the NCBI GEO database under accession number GSE293991.

### Validation of RNA sequencing by qRT-PCR

Ten genes identified as significantly up- or down-regulated were selected to verify the reliability of the RNA sequencing data. qRT-PCR was performed as described [40] using a Mx3000P qPCR cycler (Agilent Technologies, Santa Clara, CA, USA). Primers are summarized in Supplementary Table S1. Briefly, expression was quantified by SYBR Green-based qRT-PCR using the same RNA samples that were subjected to RNA sequencing. cDNA was synthesized using RevertAid Premium Reverse Transcriptase (Thermo Fisher Scientific GmbH, Dreieich, Germany) and primed with random hexamer primers (N6) (Thermo Fisher Scientific GmbH, Dreieich, Germany). Each reaction was performed in triplicate using SYBR Green and DreamTaq^TM^ Hot Start DNA Polymerase (Thermo Fisher Scientific GmbH, Dreieich, Germany) under standard cycling conditions (95°C for 10 min, followed by 40 cycles of 95°C for 15 s and 60°C for 1 min), followed by a melting-curve analysis to confirm product specificity. The housekeeping genes *YWHAZ* and *RPLP0*, previously validated as stable reference genes [41], were used for normalization. Their stability was assessed based on the Ct values of technical triplicates. For both genes across all conditions, variations < 0.5 Ct were observed, indicating stable expression across all experimental conditions. In addition, an internal standard (GAPDH, 20 ng control DNA) was included in triplicate on each plate to test inter-plate variation. The inter-plate deviation was ~0.27 Ct, confirming high technical reproducibility. Relative gene expression levels were calculated using the 2^−ΔΔCt^ method [42].

### Determination of cell death markers

To evaluate toxin-induced cell death in co-cultured Caco-2/HT29-MTX cells (differentiated for 28 d), three complementary luminescence-based assays were employed. The Caspase-Glo® 1 Inflammasome Assay (Promega, Walldorf, Germany) was used for pyroptosis, detecting inflammasome-associated caspase activation characteristic of inflammatory cell death [43]43. Quantification after 1 h was chosen to capture an early inflammasome-/pyroptosis-associated response. The Caspase-Glo® 3/7 Assay (Promega, Walldorf, Germany) quantified executioner caspase activity, serving as a marker of apoptosis and reflecting the activation of programmed cell death pathways [44,45]. Caspase-3/7 activity was measured after 3 h as an intermediate marker at the beginning of cell death. In parallel, the LDH-Glo® Cytotoxicity Assay (Promega, Walldorf, Germany) was used to measure lactate dehydrogenase release as an indicator of necrosis or late-stage membrane damage [46]. Previous monitoring of cell morphology by microscopy determined 6 h as optimum measurement time. All assays were conducted according to the manufacturer’s protocols. Measurements were performed using an Infinite® 200 plate reader (Tecan, Männedorf, Switzerland) with an integration time of 1000 ms. Untreated cells served as the negative controls. For the LDH cytotoxicity control, cells were lysed with 2 % Triton X-100 for 1 h and Ac-YVAD-CHO was used as a specific inhibitor of caspase-1. Data was normalized to the control of each experiment.

### Statistical analysis

All quantitative data are presented as mean ± standard deviation (SD). The experiments were performed using three independent biological replicates with technical triplicates each, unless stated otherwise. Graphs were generated using Microsoft Excel or GraphPad Prism version 10.0.0 for Windows, GraphPad Software, Boston, Massachusetts USA, www.graphpad.com. Statistical significance was assessed in GraphPad Prism. For this, normal distribution of the data was evaluated using the D’Agostino-Pearson test and by visual inspection of residual quantile–quantile (Q–Q) plots. Datasets with two factors and paired measurements were analyzed using two-way repeated-measures ANOVA, followed by Holm-Sidak multiple-comparisons testing. For normally distributed unpaired data, ordinary one-way or two-way ANOVA was applied; Tukey’s post hoc test was used for all pairwise comparisons, whereas Holm-Sidak correction was used when only selected comparisons were performed. For non-normally distributed data, the Kruskal–Wallis test was used, followed by Dunn’s multiple comparisons test. Adjusted *p* ≤ 0.05 was considered statistically significant. Asterisks in figures denote adjusted *p*-values. ns: not significant; *: *p* < 0.05; **: *p* < 0.01; ***: *p* < 0.001; ****: *p* < 0.0001.

RNA sequencing data were assessed with DESeq2 [47], and *p*-values were adjusted using the Benjamini–Hochberg method [48]. Genes with adjusted *p*-value < 0.05 and log_2_ fold change > 1 were defined as significantly differentially expressed genes (DEGs). Pre-ranked Gene Set Enrichment Analysis (GSEA) [49] was performed using the Molecular Signatures Database (MSigDB) Hallmark gene sets (1,000 permutations). Gene identifiers were mapped using the MSigDB Human Ensemble Gene ID chip annotation (v2025.1), and enrichment was tested against h.all. v2025.1. Hs Hallmark gene sets [50,51], with False Discovery Rate (FDR) q-value < 0.25. Kyoto Encyclopedia of Genes and Genomes (KEGG) pathway enrichment analysis of up to 20 pathways with FDR < 0.05 was performed using Shiny GO v0.85.1 [52].

## Results

### Comparable binding capacity of Hbl B to Caco-2, HT29-MTX and a 9:1 co-culture

**Figure 1.**
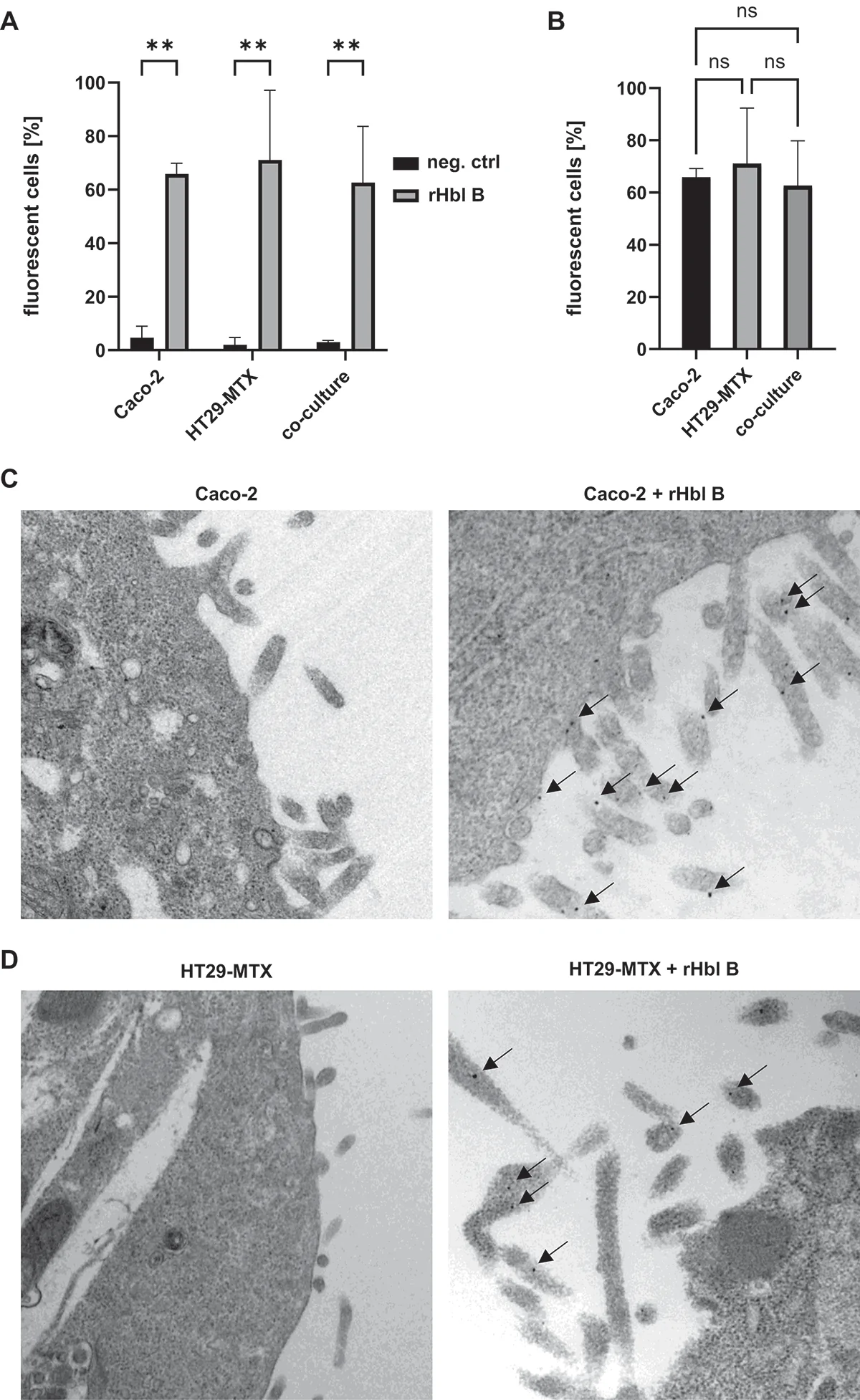
Binding of rHbl B to Caco-2 and HT29-MTX cells. A. Quantitative comparison of the two cell lines and the co-culture to their corresponding negative controls (both antibodies, no rHbl B) as assessed by flow cytometry using Hbl B-specific mAb 1G8 [32] and Alexa Fluor® 488-conjugated goat anti-mouse antibody. Two independent experiments were performed; each measured in triplicates. Shown is the percentage of fluorescent cells within the gated cell population. B. Comparison of the three cell models. Statistical significance was assessed using ordinary one-way ANOVA followed by Tukey’s multiple-comparisons test (B), and two-way ANOVA followed by the Holm-Sidak multiple-comparisons test (A). ns: not significant; **: *p* < 0.01. C. Localization of rHbl B on the surface of Caco-2 cells. Samples were analysed by transmission electron microscopy using Hbl B-specific mAb 1G8 [32] and goat anti-mouse IgG-gold conjugate. Left image: negative control (original magnification 20.000x). Right image: rHbl B (original magnification 21.560x). D. Analogous localization of rHbl B on the surface of HT29-MTX cells. Left image: negative control (original magnification 20.000x). Right image: rHbl B (original magnification 27.000x). Arrows point out some representative rHbl B binding sites.

Flow cytometry was used to quantitatively evaluate the binding of the enterotoxins to three intestinal epithelial models. Due to the well-established examination procedure on Vero cells [8,15], rHbl B was selected as an example to investigate putative differences between cell lines. In comparison, HT29-MTX cells showed the highest proportion of rHbl B-positive cells (71.06%), followed by Caco-2 (65.85%), and the co-culture (62.65%). However, statistically significant differences were not detected (Figure 1(A, B)). In order to provide additional visualization of rHbl B attachment to the two target cell lines, rHbl B was localized on the surface of Caco-2 (Figure 1(C)) and HT29-MTX cells (Figure 1(D)) via immunogold transmission electron microscopy. rHbl B was present at the cell membranes, primarily in the microvilli region. It can therefore be concluded that rHbl B binds with comparable affinity to both Caco-2 and HT29-MTX cells.

### Cell line- and enterotoxin-dependent cytotoxic effects

**Figure 2.**
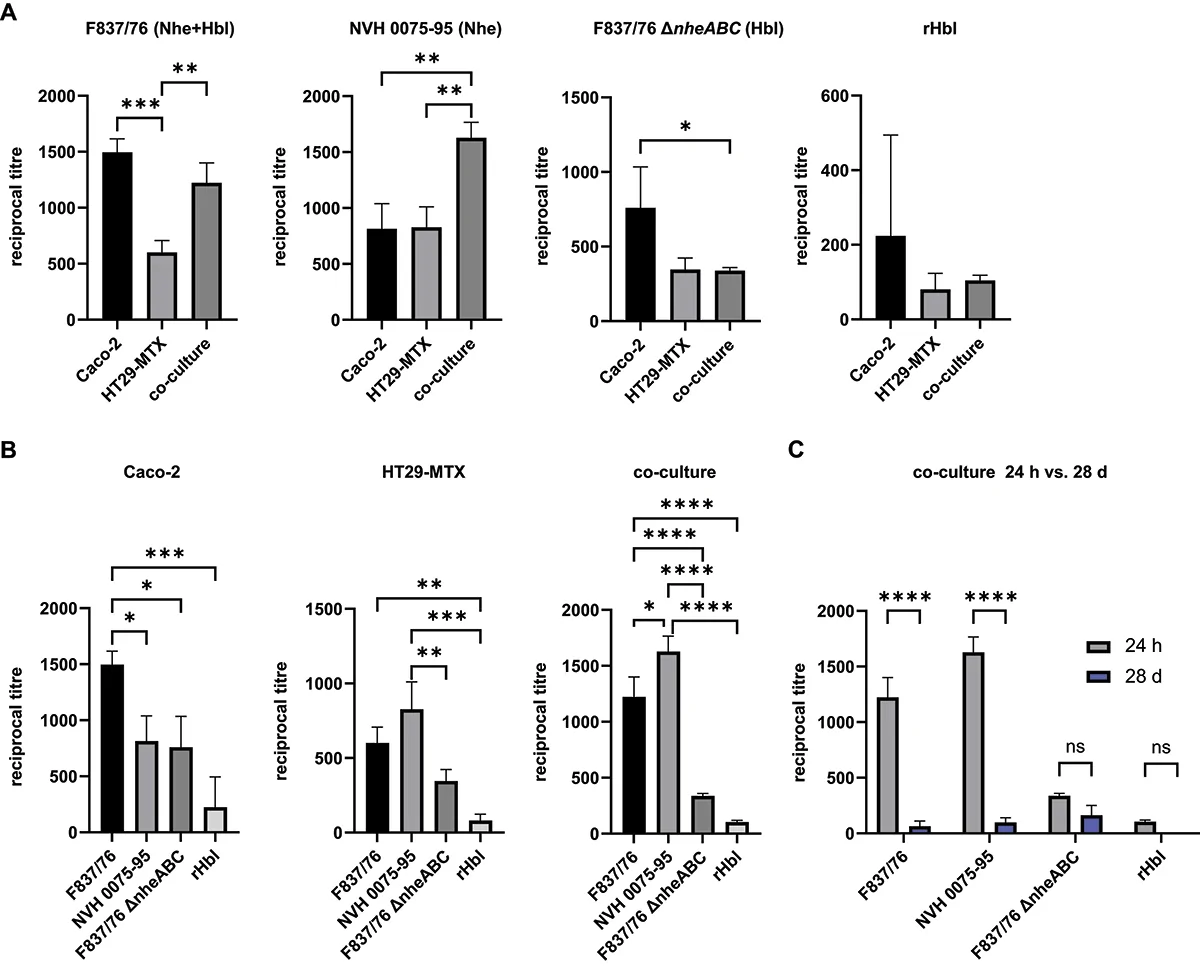
Comparison of reciprocal IC_50_ values of *B. cereus* enterotoxins toward Caco-2, HT29-MTX and the Caco-2/HT29-MTX co-culture. Cytotoxicity was assessed by WST-1 bioassays, where undifferentiated cells were exposed for 24 h to serial dilutions of culture supernatants from strains F837/76 (Nhe+Hbl), NVH 0075–95 (Nhe), F837/76 Δ*nheABC* (Hbl), as well as rHbl. A. Cell line comparison for each toxin preparation. B. Toxin comparison within each cell model. C. Comparison of reciprocal titers determined on the co-culture. Gray: cultivated for 24 h before toxin exposure (as in B). Blue: cultivated for 28 d (differentiated) before toxin exposure. Statistical significance was assessed using ordinary one-way ANOVA followed by Tukey’s multiple-comparisons test (A, B), and two-way ANOVA followed by the Holm-Sidak multiple-comparisons test (C) for normally distributed data. For non-normally distributed data, the Kruskal–Wallis test was used, followed by Dunn’s multiple comparisons test. ns: not significant; *: *p* < 0.05; **: *p* < 0.01; ***: *p* < 0.001; ****: *p* < 0.0001.

In WST-1 cell viability assays (Figure 2), *B. cereus* strain F837/76 (Nhe+Hbl) showed significantly higher toxicity toward Caco-2 cells and the co-culture than to HT29-MTX. The corresponding Δ*nheABC* strain (Hbl) exhibited generally lower toxicity, with Caco-2 cells still being more sensitive that HT29-MTX. Surprisingly, similar reciprocal IC_50_ titers were determined for NVH 0075–95 (Nhe) on both cell lines, with a significant increase on the co-culture. Previously conducted enzyme immunoassays showed that all three strains produced comparable amounts of Hbl and/or Nhe (Supplementary Fig. S1). For comparison, recombinant Hbl toxin was also included, which predictably had a significantly weaker effect, with no distinct differences between the cell culture models (Figure 2(A,B)). These findings show that both toxin composition and epithelial cell type influence the level of effectiveness of the enterotoxins. Differentiation of the cell monolayer also plays a decisive role, as shown by the comparison of the Caco-2/HT29-MTX co-culture after 24 h and after 28 d of cultivation. A significantly higher sensitivity of the 24 h culture was determined toward Nhe/Hbl and Nhe, but interestingly not to Hbl alone (Figure 2(C)). Additionally, next to IC_50_, the lethal (IC_90_) and sublethal (IC_10_) concentrations of each *B. cereus* supernatant were determined for application in subsequent experiments. Viability assay data were supported by live-cell imaging. Morphological changes (decrease in cell size, density and coverage) and cell death were observed to occur over time (Supplementary recordings S1-6). To sum up, Hbl seems to have a higher impact on Caco-2, and Nhe on HT29-MTX cells. 24 h cell cultures were significantly more susceptible than 28 d differentiated cells.

### Cytotoxicity due to interference of the enterotoxins with membrane stability and ion transport

**Figure 3.**
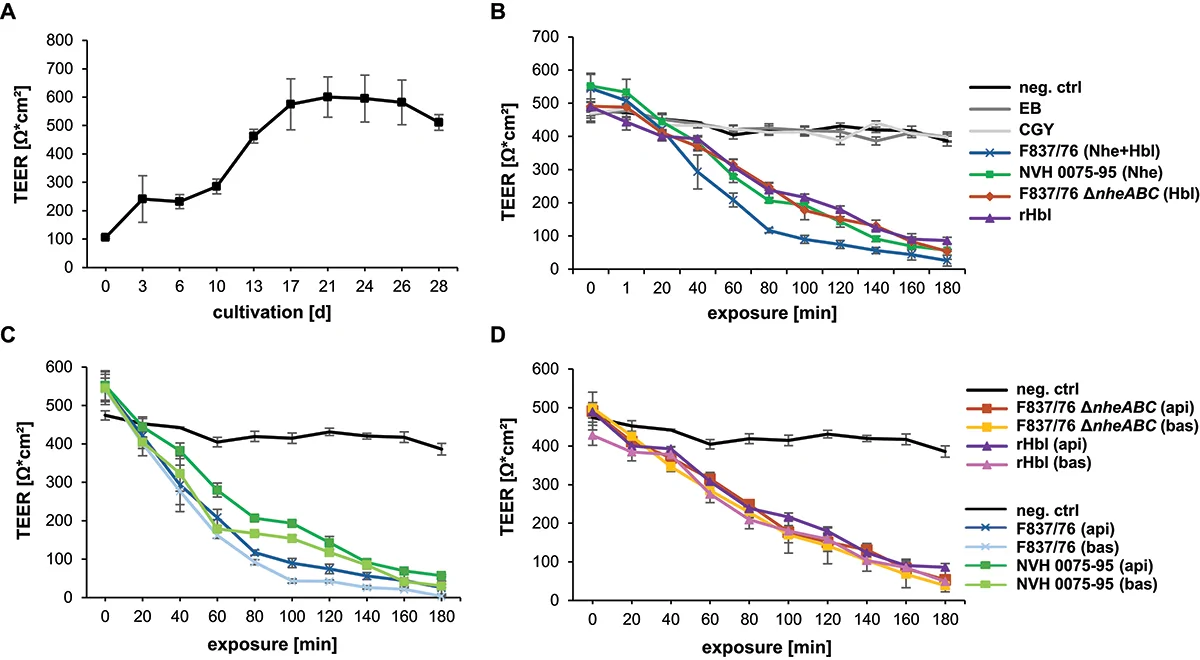
Results of transepithelial electrical resistance (TEER) measurements on the Caco-2/HT29-MTX co-cultures maintained in Snapwells®. A. Increase in TEER values over a cultivation period of 28 d. B. Time-dependent changes in TEER of 21 d co-cultures following apical exposure to *B. cereus* enterotoxins (1:40 dilution). C. and D. Side-dependent effects of toxin exposure on epithelial integrity at a 1:40 dilution. TEER was measured every 20 min using an EVOM^2^ voltohmmeter. Experiments were performed twice with triplicate wells; one representative run is shown because baseline TEER varied between runs and strongly influenced absolute resistance values. neg. ctrl: untreated cells, CGY: bacterial culture medium, EB: elution buffer used for rHbl, api: apical site exposure, bas: basolateral site exposure.

The pore-forming *B. cereus* enterotoxins exhibit cytotoxic activity by disrupting cell membrane integrity. This has been shown on planar lipid bilayers and on different cell lines, but not yet for the Caco-2/HT29-MTX co-culture model. In the following, this study focuses solely on the latter and no longer on the comparison of the two cell lines. During cultivation of this model, transepithelial electrical resistance (TEER) was measured every 2–3 d to monitor monolayer formation and barrier integrity. A gradual increase in TEER values was observed over time, reaching a maximum after approx. 21–24 d (Figure 3(A)). Cells between 21 and 28 d of differentiation were selected for subsequent experiments, representing a stable and functionally mature epithelial barrier. To demonstrate the effect of the *B. cereus* enterotoxins on epithelial barrier integrity and cell–cell contact of the Caco-2/HT29-MTX model, TEER measurements were performed during a 3 h toxin exposure. Negative controls (untreated cells and media/buffers) were included for comparison. Strongest time-dependent decrease of TEER values was observed for the Nhe+Hbl producing strain F837/76, followed by NVH 0075–95 (Nhe), F837/76 Δ*nheABC* (Hbl), and rHbl (Figure 3(B)). To evaluate polarity-dependent effects, the toxins were applied either to the apical or basolateral compartment of the monolayer. TEER values decreased overtime under both conditions. Basolateral exposure caused a slightly, but not statistically significant greater reduction than apical treatment (Figure 3(C,D)).

**Figure 4.**
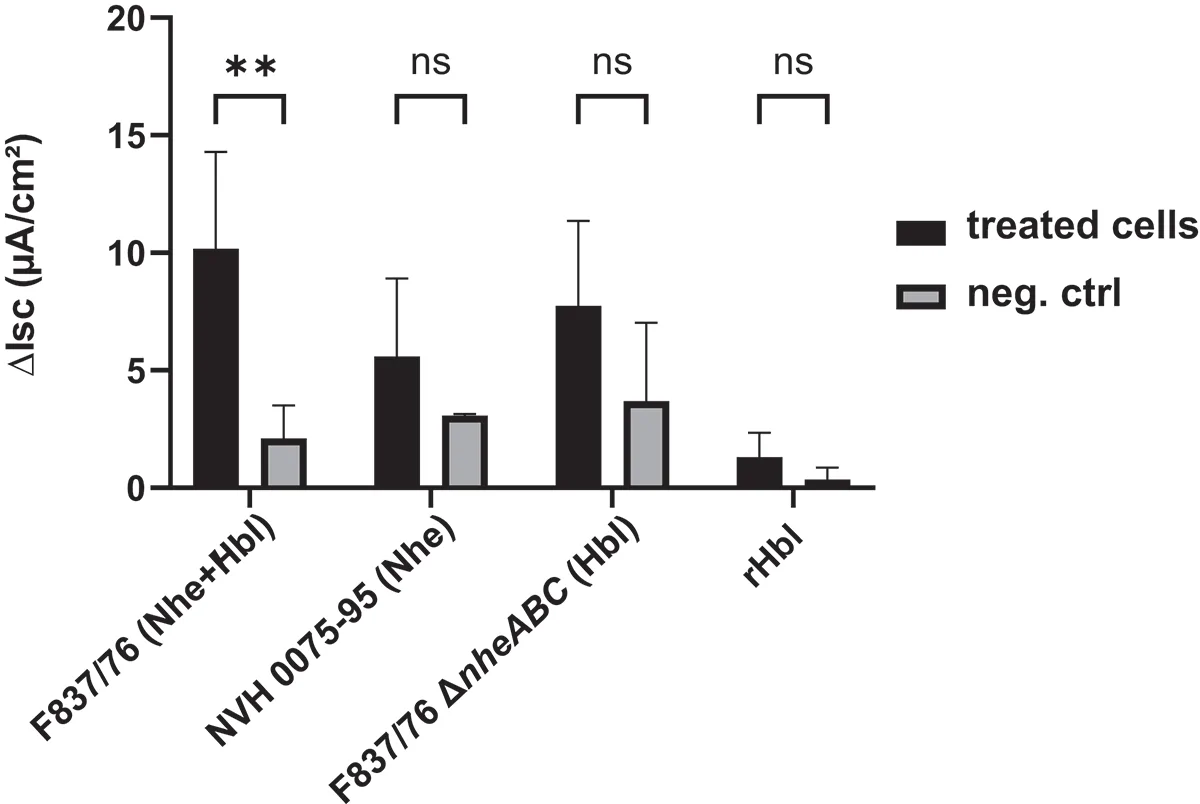
Change in short-circuit current (ΔIsc) in Caco-2/HT29-MTX co-cultures after 2.5 h of toxin exposure. Enterotoxin-containing *B. cereus* supernatants and rHbl were added to the apical side of the Ussing chamber at a 1:40 dilution. Bacterial culture medium CGY or rHbl elution buffer was applied as corresponding negative control. The chamber was maintained at 37°C using a water bath, and carbogen was continuously supplied to homogenize the buffer and support cell viability. ΔIsc was calculated as the difference between maximal and basal Isc. Basal Isc (before treatment) was defined as the mean of 10 measurements immediately before toxin addition, and maximal Isc (after treatment) as the highest value recorded during the 2.5 h exposure period. *p*-values indicate statistically significant differences in ΔIsc between treated cells and the negative control. Statistical significance was assessed using a two-way ANOVA followed by Holm-Sidak multiple comparisons correction. ns: not significant; **: *p* < 0.01.

The effect of the enterotoxins on ion transport in Caco-2/HT29-MTX co-cultures was assessed in Ussing chambers during 2.5 h of toxin exposure. In contrast to TEER, this is performed under controlled electrophysiological conditions throughout the duration of the measurement [35–37]. First, transepithelial resistance (Rt) is determined which indicates electrical barrier (tight junction and paracellular) integrity similar to TEER measurements. Second, transepithelial current (Isc) is used to measure electrogenic ion transport which means active/secretory transport mechanisms rather than barrier damage [37]. Isc increased after exposure to all three *B. cereus* culture supernatants compared to the corresponding negative controls/baseline values. The strongest and significant response was observed for strain F837/76 (Nhe+Hbl), whereas NVH 0075–95 (Nhe) and F837/76 Δ*nheABC* (Hbl) induced a more moderate increase. In contrast, rHbl caused only minimal changes in short-circuit current (Figure 4 and Supplementary Fig. S2). In parallel with the toxin-induced increase in Isc, Rt gradually decreased over time; however, this decrease was relatively slow compared with the pronounced Rt/TEER drop measured using the EVOM^2^ system. In accordance with rising Isc, Rt decreased significantly under Nhe+Hbl treatment (Supplementary Fig. S3). In conclusion, epithelial barrier integrity was disrupted and electrogenic ion transport was enhanced by all applied *B. cereus* toxins, while Nhe+Hbl exhibited the strongest effect.

### Partial recovery of the co-cultures after enterotoxin exposure

**Figure 5.**
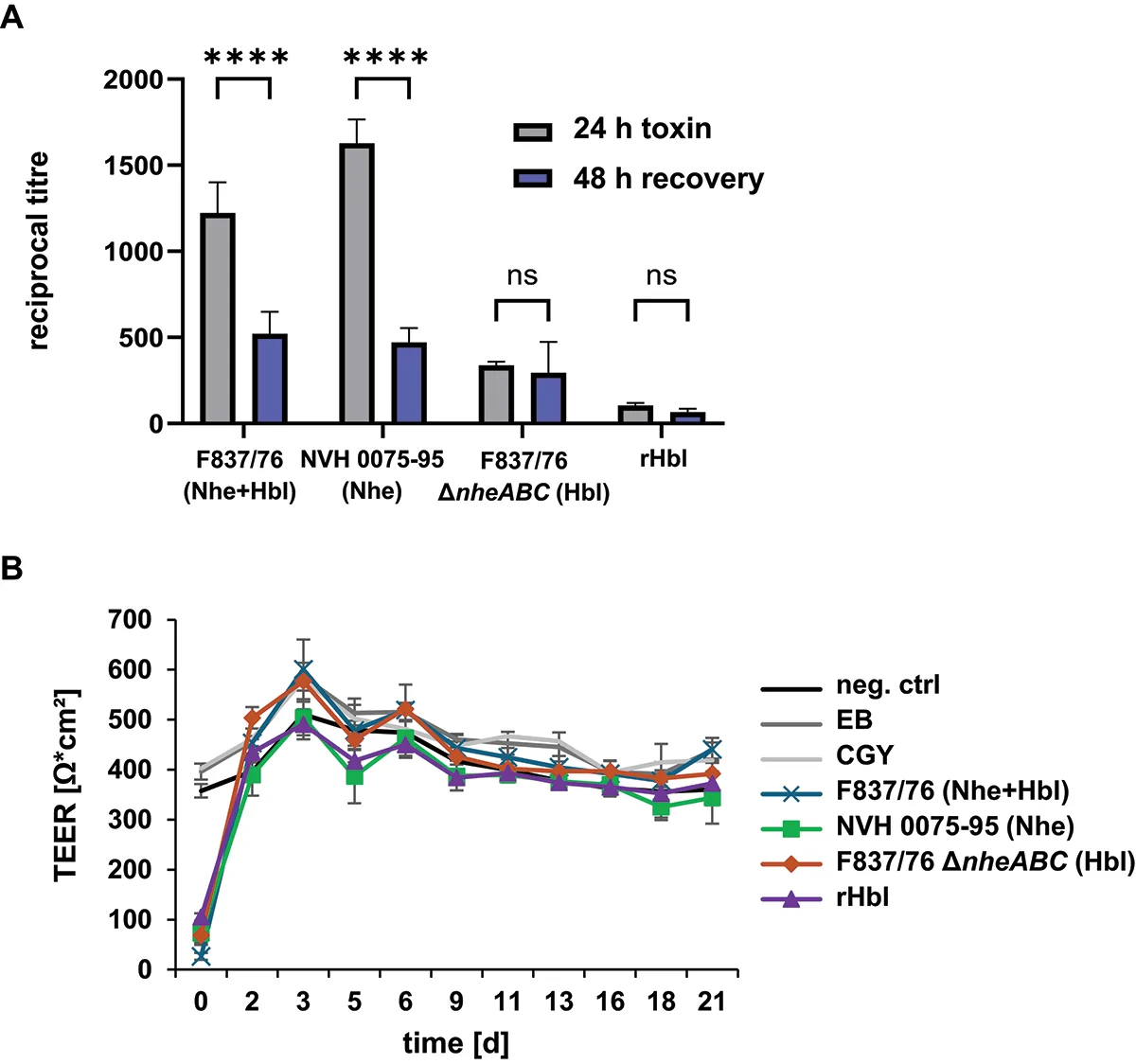
Restoration of cell viability and epithelial barrier function after toxin exposure. A. Comparison of reciprocal titers derived from WST-1 viability assays in Caco-2/HT29-MTX co-cultures (non-differentiated) after 24 h toxin exposure and after toxin removal followed by an additional 48 h recovery period in toxin-free medium. Statistical significance was assessed using two-way ANOVA followed by the Holm-Sidak multiple-comparisons test. ns: not significant, ****: *p* < 0.0001. B. Results of TEER measurements on the Caco-2/HT29-MTX co-cultures further cultivated in Snapwells® after toxin exposure (see Figure 3(B)) and removal. TEER was measured every second day using an EVOM^2^ voltohmmeter. Experiments were performed twice with triplicate wells. neg. ctrl: untreated cells, CGY: bacterial culture medium, EB: elution buffer used for rHbl.

Similar to the WST-1 cell viability assays described above, it was evaluated whether the Caco-2/HT29-MTX co-cultures (non-differentiated, 24 h cultures) were able to recover from enterotoxin exposure. Enterotoxin-specific reciprocal titers were obtained after 24 h toxin exposure (compare above). After removal of the supernatant (including toxins, dead cells, cell debris) and additional 48 h incubation in toxin-free medium, titers were determined again, which were significantly lower for F837/76 (Nhe+Hbl) and NVH 0075–95 (Nhe) than after 24 h toxin application (Figure 5(A)). This indicates an enhancement of mitochondrial activity, which corresponds to an increasing number of viable cells. The differences between the titers obtained after 24 h toxin exposure and 48 h additional incubation time for F837/76 Δ*nheABC* (Hbl) and rHbl were not significant as the initial effect of the applied toxins was comparably weak (Figure 5(A)). These observations were again supported by live-cell imaging after application of low toxin concentration (F837/76 (Nhe+Hbl): 1:600; NVH 0075–95 (Nhe) and F837/76 Δ*nheABC* (Hbl) 1:400). Cells displayed partial recovery as evidenced by gradual reattachment, spreading, and regrowth over 48 h after toxin removal, with one image taken every 10 min (Supplementary recordings S7-9). Moreover, TEER values, i.e. barrier integrity of differentiated, 21 d co-cultures could be restored after removal of apically applied toxins (see Figure 3(B)) and subsequent cultivation in growth medium in the Snapwells® for several days (Figure 5(B)). To sum up, recovery of cell viability, growth and epithelial barrier integrity was detected, regardless of the applied enterotoxin.

### Time- and dose-dependent transcriptional response of the co-culture to enterotoxin exposure

**Figure 6.**
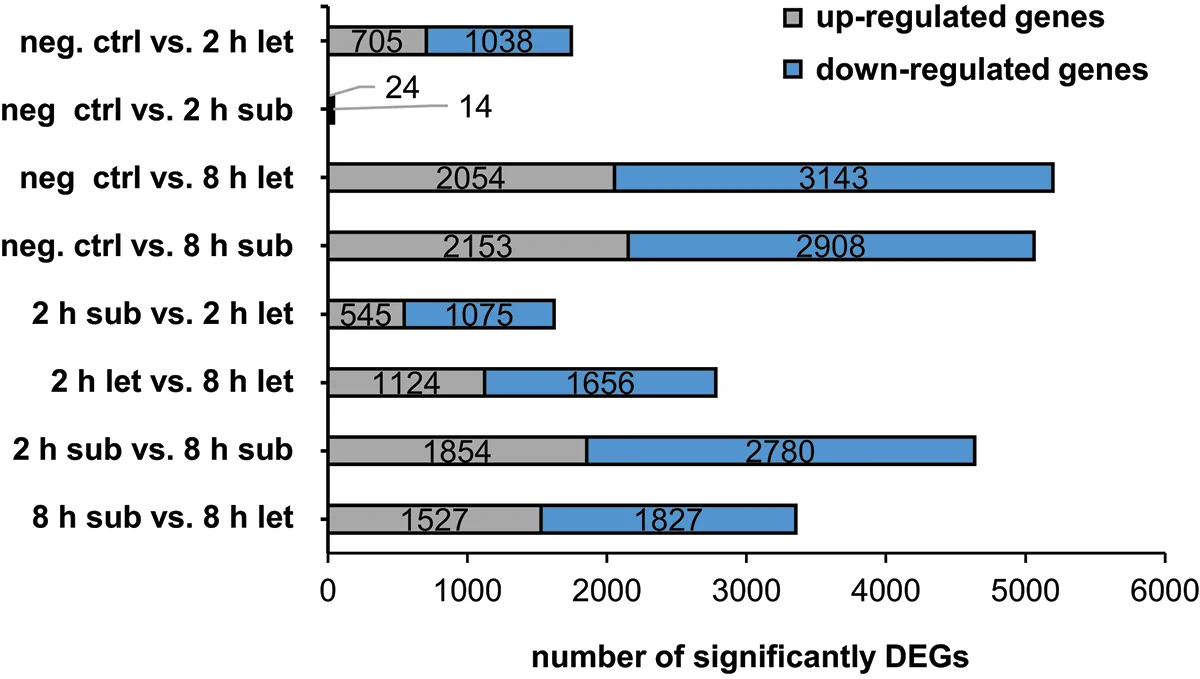
Differential gene expression in Caco-2/HT29-MTX co-cultures exposed to the supernatant of *B. cereus* strain F837/76 (Nhe+Hbl). Different exposure times (2 and 8 h) and different toxin doses (lethal and sublethal) were compared to the untreated negative control as well as to each other. Bars represent the number of significantly up-regulated (grey) or down-regulated (blue) differentially expressed genes (DEGs) in the respective latter condition, defined by adjusted *p*-values < 0.05 and log_2_ fold change > 1.

So far, it was shown that the *B. cereus* enterotoxins bind and interfere with membrane integrity which ultimately leads to death of the Caco-2/HT29-MTX co-culture model. It was further seen that the cells are able to (partially) recover from toxin exposure under appropriate circumstances. The final part of this study focused on uncovering underlying mechanisms and pathways. Thus, the transcriptional response of the co-culture to F837/76 (Nhe+Hbl) was investigated via RNA sequencing. For this, 28 d differentiated monolayers were exposed to lethal (IC_90_; let) and sublethal (IC_10_; sub) toxin doses both for 2 and 8 h. Untreated cells were included as negative control. Initial differential gene expression analysis was performed by Azenta Life Sciences. Genes with an adjusted *p* < 0.05 and an absolute log_2_ fold change > 1 between conditions were defined as significantly differentially expressed genes (DEGs). As summarized in Fig. 6 and Supplementary Fig. S4, the largest number of DEGs was detected at 8 h under lethal exposure, followed by 8 h under sublethal exposure. In contrast, 2 h sublethal exposure caused only minor changes compared to non-treated cells. Across comparisons, downregulated genes exceeded upregulated genes, indicating a predominant suppression of gene expression under F837/76 (Nhe+Hbl) toxin exposure (Figure 6). Supplementary Tables S2 and S3 summarize the top 30 differentially expressed genes among all conditions compared. In conclusion, the Caco-2/HT29-MTX co-culture responds with varying strength to Nhe+Hbl concentrations and exposure times.

### *B. cereus* enterotoxins induce inflammation- and stress-associated pathways

**Figure 7.**
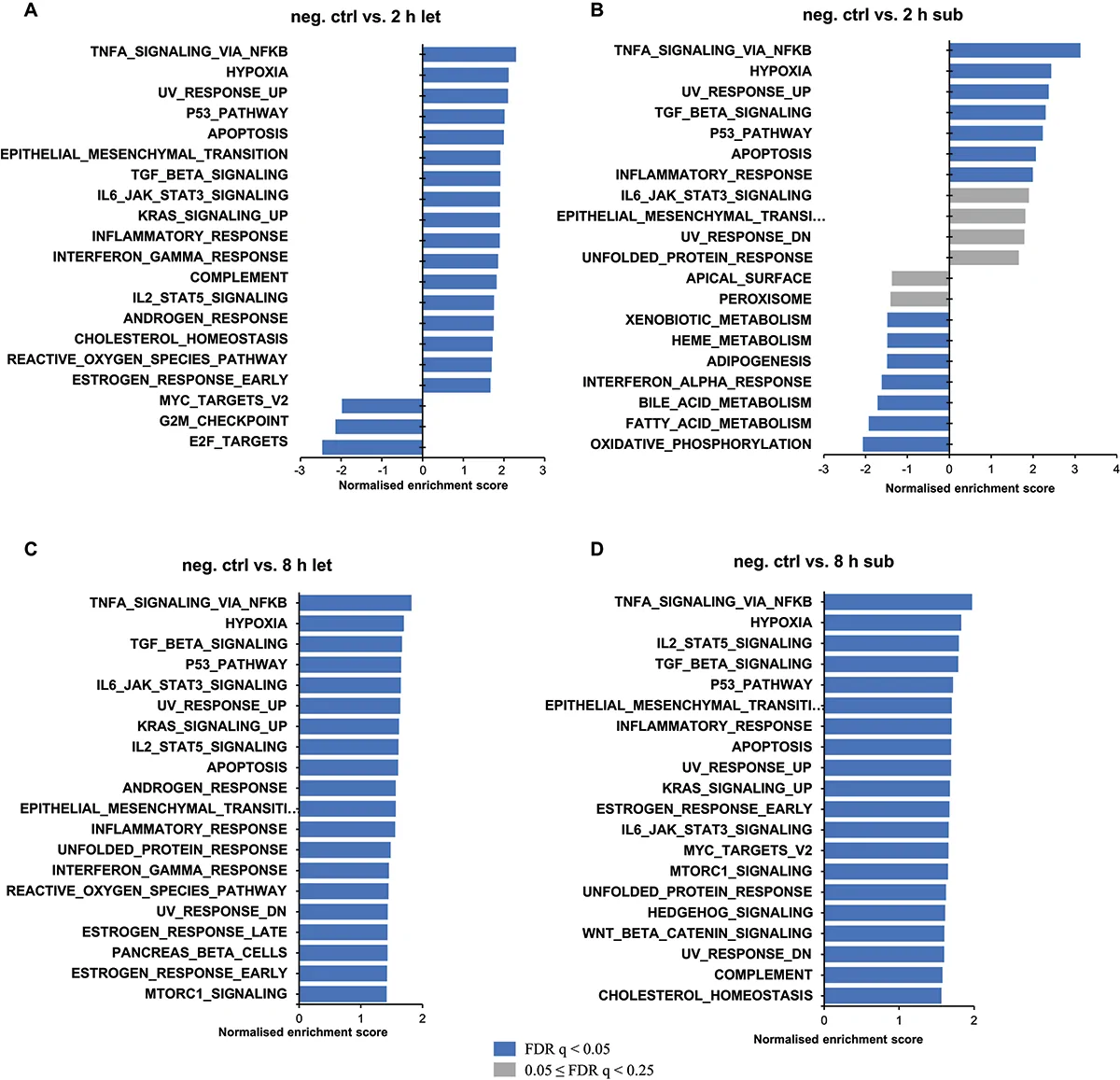
Pre-ranked Gene Set Enrichment Analysis (GSEA) of MSigDB Hallmark gene sets comparing cells exposed to strain F837/76 (Nhe+Hbl) with untreated cells (neg. ctrl). Conditions were A. 2 h lethal dose, B. 2 h sublethal dose, C. 8 h lethal dose, D. 8 h sublethal dose. Genes were ranked using the score -log_10_(padj) × sign (log_2_ fold change). Results indicate the direction of differential expression between toxin-treated and untreated cells. The 20 most enriched Hallmark pathways are shown, sorted by normalized enrichment score (NES). Positive NES-values indicate pathways enriched in toxin-treated cells; negative NES-values indicate pathways enriched in untreated cells. Blue bars represent gene sets with FDR q < 0.05, gray bars represent 0.05 ≤ FDR q < 0.25. FDR q is a multiple-testing-adjusted *p*-value that controls the False Discovery Rate (FDR). Examples of GSEA enrichment plots are shown in Supplementary Fig. S5.

To characterize pathway-level responses of the Caco-2/HT29-MTX co-culture model toward Nhe+Hbl enterotoxin exposure, pre-ranked Gene Set Enrichment Analysis (GSEA) using the MSigDB Hallmark gene set collection was performed [49–51]. Pre-ranked GSEA of MSigDB Hallmark gene sets revealed consistent enrichment of inflammation- and stress-associated pathways across toxin exposure conditions, with clear dose- and time-dependent differences. The strongest positively enriched Hallmark gene sets (FDR q-values < 0.05) included TNFα signaling via NF-κB, hypoxia, p53 pathway, apoptosis, TGF-β signaling, IL6–JAK–STAT3 signaling, epithelial–mesenchymal transition (EMT), and inflammatory response (Figure 7(A-D)). Condition-specific patterns were also visible. Under 2 h lethal exposure, proliferation and cell-cycle programs (E2F targets, G2M checkpoint, and MYC targets V2) were negatively enriched, indicating early inhibition of cell proliferative signaling (Figure 7(A)). Under 2 h sublethal toxin exposure, several metabolism-related gene sets, including oxidative phosphorylation, fatty acid metabolism, and xenobiotic metabolism, showed negative enrichment (Figure 7(B)). Across exposure times, several transcripts showed enhancement at the later time point, such as MT-TG and GEM (Supplementary Tables S2 and S3), consistent with progressive stress accumulation and ongoing remodeling. Examples for positively and negatively enriched Hallmark gene sets are depicted as GSEA enrichment plots in Supplementary Figure S5. Furthermore, the top 20 leading-edge (driver) genes contributing to the identification of the six most significantly enriched Hallmark gene sets are summarized in Table 1.

**Table 1.** Top 20 leading-edge (driver) genes from the six most significantly enriched Hallmark gene sets identified by GSEA in the Caco-2/HT29-MTX co-culture model exposed to supernatant of strain F837/76 (Nhe+Hbl) for 2 h at a lethal dose, compared to untreated cells. Nearly all of these genes were up-regulated. Here, only the 2 h lethal condition data is shown, as most of the processes and signaling pathways also observed after 8 h were already activated at this point.

TNFα via NF-κB	Hypoxia	p53 pathway	Apoptosis	Inflammatory response	IL6-JAK-STAT3 signalling
*JUN*	*JUN*	*JUN*	*JUN*	*NFKBIA*	*MAFF*
*CXCL2*	*TNFAIP3*	*FOS*	*ATF3*	*CCL20*	*BMP2*
*RCAN1*	*FOS*	*PPP1R15A*	*BMP2*	*HBEGF*	*MAP3K8*
*NFKBIA*	*PPP1R15A*	*ATF3*	*IER3*	*KLF6*	*NFKBIZ*
*TNFAIP3*	*ATF3*	*BMP2*	*SLC20A1*	*BTG2*	*ARL4A*
*FOS*	*DUSP1*	*IER3*	*GADD45A*	*ICAM1*	*KLF6*
*PPP1R15A*	*MAFF*	*GADD45A*	*SAT1*	*CCL2*	*SPRY4*
*ATF3*	*ZFP36*	*EPHA2*	*BTG2*	*LDLR*	*GADD45B*
*DUSP1*	*IER3*	*SAT1*	*BIRC3*	*MXD1*	*MXD1*
*JUNB*	*PCK1*	*IER5*	*MCL1*	*IRF1*	*PLIN2*
*EGR1*	*PNRC1*	*HBEGF*	*SQSTM1*	*NAMPT*	*RHOB*
*CXCL3*	*TIPARP*	*BTG2*	*GADD45B*	*EREG*	*EMP1*
*NR4A1*	*KLF6*	*SERTAD3*	*IRF1*	*CD55*	*PNP*
*MAFF*	*NOCT*	*MXD1*	*RHOB*	*ITGB8*	*BHLHE40*
*GEM*	*CCN1*	*RNF19B*	*EMP1*	*SLC11A2*	*NDRG1*
*ZBTB10*	*PLIN2*	*ZFP36L1*	*EGR3*	*ADM*	*RORA*
*ZFP36*	*GCNT2*	*TRAF4*	*SMAD7*	*CDKN1A*	*GABARAPL1*
*BMP2*	*JMJD6*	*MDM2*	*EREG*	*ITGA5*	*MYO1E*
*CXCL1*	*SDC4*	*TOB1*	*DDIT3*	*SGMS2*	*AGER*
*IER3*	*TGFB3*	*BTG1*	*ISG20*	*GCH1*	*IRF6*

**Figure 8.**
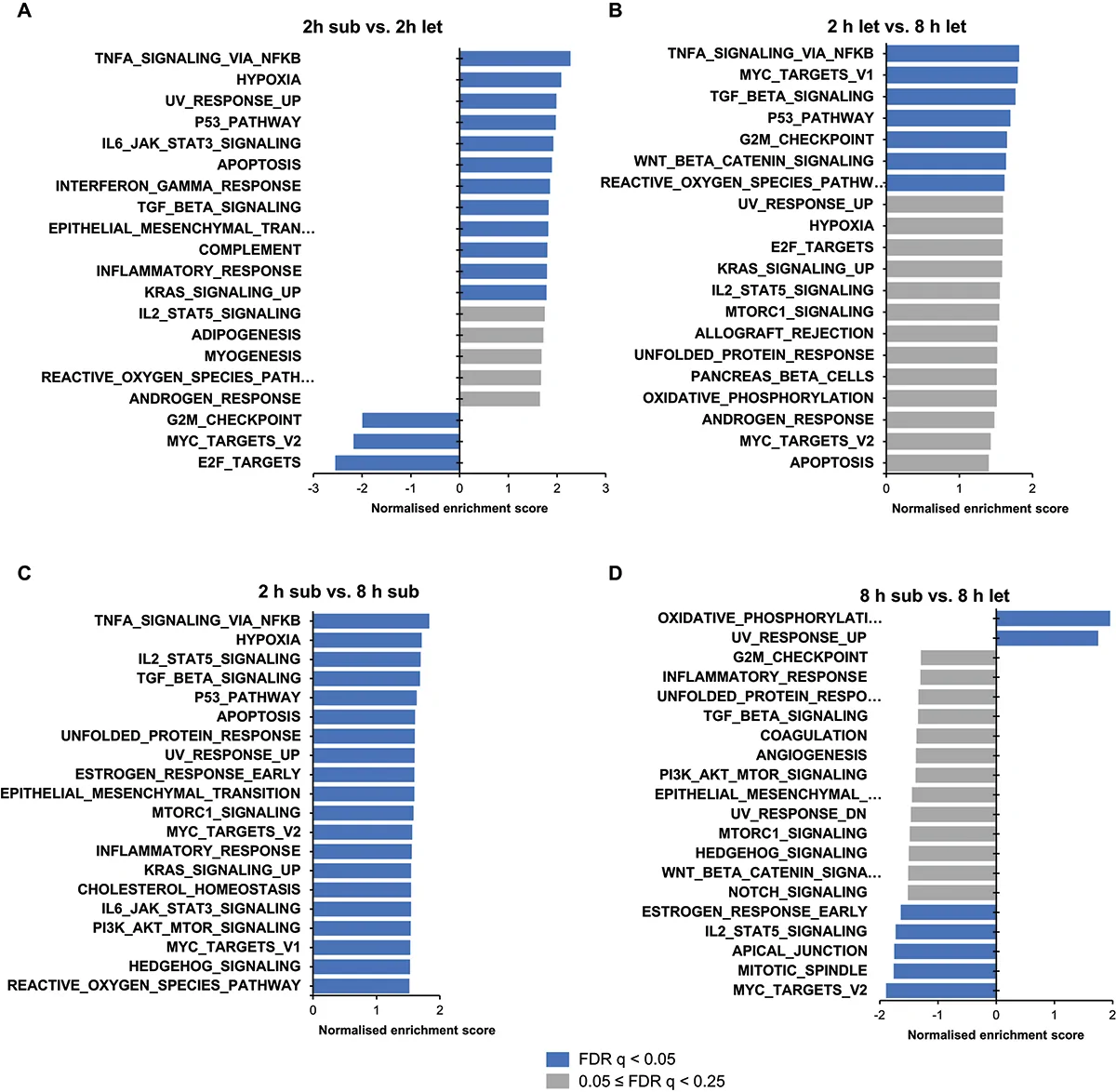
Comparison of the top 20 MSigDB Hallmark gene sets across exposure times (2 h vs 8 h) and toxin doses (lethal vs sublethal) for cells treated with strain F837/76 (Nhe+Hbl). Pairwise comparisons include A. dose effect at 2 h, B. time effect under lethal exposure, C. time effect under sublethal exposure, and D. dose effect at 8 h. For each comparison, the 20 most enriched Hallmark pathways are shown, sorted by normalized enrichment score (NES), to highlight shared and condition-specific pathway signatures. Positive NES-values indicate pathways enriched in the latter condition; negative NES-values indicate pathways enriched in the first condition. Blue bars represent gene sets with FDR q < 0.05, and grey bars represent 0.05 ≤ FDR q < 0.25.

Additionally, direct comparisons across dose (Figure 8(A,D)) and time (Figure 8(B, C)) showed that while negative enrichment of cell-cycle-related Hallmarks was more prominent under lethal conditions (Figure 8(A,D)), 2 h sublethal exposure produced stronger enrichment of inflammatory- and stress-associated Hallmarks (including TNFα signaling via NF-κB, hypoxia, p53 pathway, IL6–JAK–STAT3 signaling, apoptosis, and TGF-β signaling; Figure 8(A)). Comparisons between sublethal and lethal conditions after 8 h revealed a mixed pattern, with numerous stress and signaling programs migrating toward the deadly condition and oxidative phosphorylation being enriched toward sublethal exposure (Figure 8(D)). Overall, consistent activation of Hallmark programs linked to NF-κB/inflammation, hypoxia, p53/apoptosis, and TGF-β/EMT appeared, while toxin dose and exposure time modulate the relative contribution of cell-cycle/proliferation vs metabolic and stress-response signatures, as reflected by NES direction and FDR category.

Additionally, KEGG pathway enrichment analysis of DEGs was performed with ShinyGO v0.85.1 [52], to supplement the rank-based Hallmark GSEA (Figure 7 and 8). In summary, KEGG enrichment independently confirmed the primary Hallmark pathways such as TNFα/NF-κB-mediated inflammation, cell-fate programs involving the p53-mediated DNA-damage/stress response, MAPK-associated stress signaling, as well as regulated cell death pathways including apoptosis (Supplementary Fig. S6 and S7). Further, 10 selected DEGs were analyzed by qRT-PCR to validate the RNA sequencing results. *JUN*, *DUSP1*, *FOS*, *TNFAIP3*, *CXCL2*, *MAFF*, and *TSC22D2* were selected from the top 30 DEGs based on their relevance to *B. cereus* enterotoxin-associated responses and their consistently strong differential expression across most experimental conditions. These genes represent key regulators of stress response, inflammation, and transcriptional control. In addition, *LYZ*, *TRPV1*, and *ST3GAL3* were selected to validate down-regulation observed in the RNA sequencing analysis. qRT-PCR results (Table 2) showed a high level of agreement with RNA sequencing data in both direction and relative magnitude of gene expression changes, thereby validating the reliability of the RNA sequencing analysis. To conclude, *B. cereus* enterotoxin exposure leads to early inhibition of cell proliferation, as well as enhanced inflammatory, stress-associated and cell-death-related pathways in the Caco-2/HT29-MTX model, depending on toxin dose and exposure time.

**Table 2.** qRT-PCR validation of RNA sequencing results. Seven up-regulated genes were selected from the top 30 DEGs, and three down-regulated genes were chosen from the significantly differential expressed genes list based on an adjusted *p*-value < 0.05 and absolute log_2_ fold change > 1. For primers and PCR conditions, see Supplementary Table S1. The values presented in Table 2 are log_2_ fold change values. -: not significantly differentially expressed and/or not tested by qRT-PCR.

ID	Name	Method	neg. ctrl. vs 2 h let	neg. ctrl. vs 2 h sub	neg. ctrl. vs 8 h let	neg. ctrl. vs 8 h sub	Regulation
ENSG00000177606	*JUN*	PCR	4.97	2.26	5.38	1.91	up
		Seq	4.74	1.15	6.35	3.07	
ENSG00000120129	*DUSP1*	PCR	3.58	3.18	4.24	2.02	up
		Seq	3.63	0	4.78	3.77	
ENSG00000081041	*CXCL2*	PCR	4.96	1.77	4.2	1.71	up
		Seq	4.42	1.02	5.04	2.32	
ENSG00000170345	*FOS*	PCR	4.45	2.96	5.14	2.54	up
		Seq	4.32	1.69	5.62	3.58	
ENSG00000196428	*TSC22D2*	PCR	1.14	1.31	1.32	1.13	up
		Seq	1.81	0	2.84	2.03	
ENSG00000118503	*TNFAIP3*	PCR	2.89	1.62	2.68	1.48	up
		Seq	2.95	0	3.79	2.72	
ENSG00000185022	*MAFF*	PCR	2.43	3.43	2.95	2.78	up
		Seq	3.12	0	4.1	4.13	
ENSG00000090382	*LYZ*	PCR	−	−	−	−2.18	down
		Seq	−1.75	−	−	−1.99	
ENSG00000196689	*TRPV1*	PCR	−	−	−1.43	−	down
		Seq	−	−	−1.6	−1.93	
ENSG00000126091	*ST3GAL3*	PCR	−	−	−1.14	−	down
		Seq	−	−	−1.95	−1.07	

### Induction of apoptosis and membrane damage by the enterotoxins

**Figure 9.**
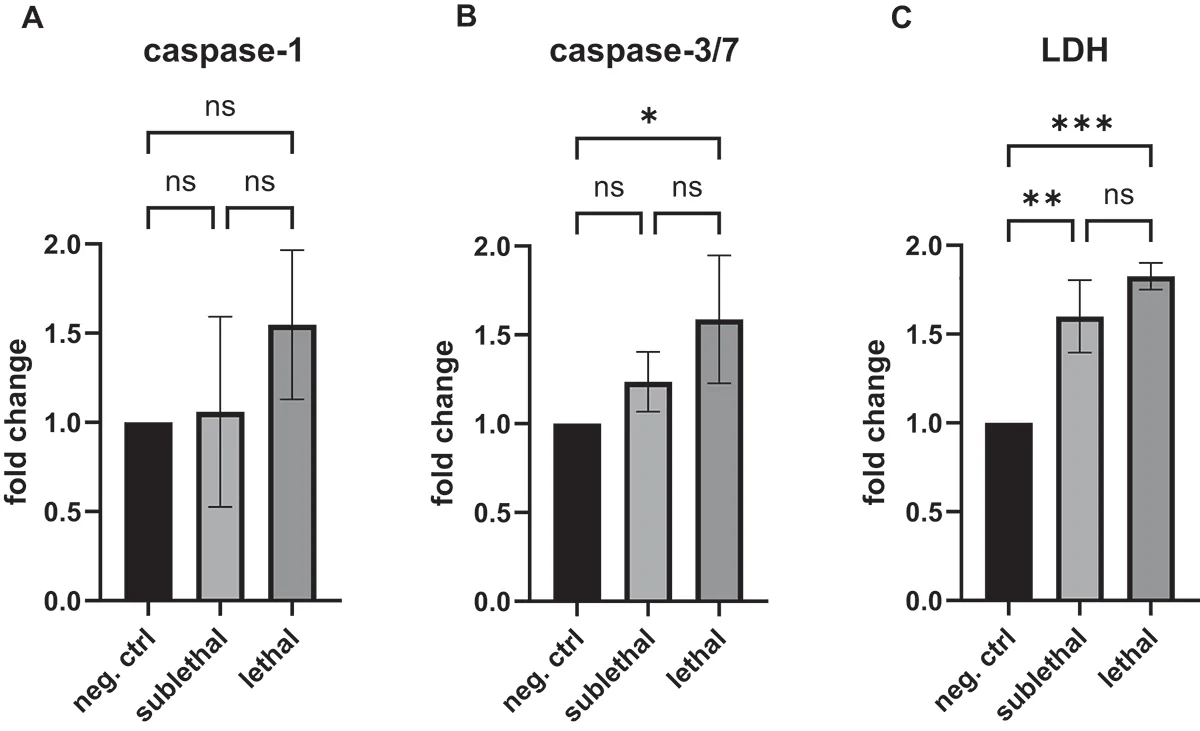
Molecular cell-death readouts after enterotoxin exposure. Differentiated Caco-2/HT29-MTX co-cultures were exposed to F837/76 supernatant (Nhe+Hbl) in sublethal (IC_10_) or lethal (IC_90_) doses. A. Luminescent caspase-1 assay was applied after 1 h. B. Caspase-3/7 assay was applied after 3 h. C. LDH assay was applied after 6 h. Results are shown as fold change relative to the negative control (untreated cells). Statistics: one-way ANOVA followed by Tukey’s multiple-comparisons test. ns: not significant; *: *p* < 0.05; **: *p* < 0.01; ***: *p* < 0.001.

To complement transcriptome analyses and to assess toxin-induced cell death signaling, caspase-1 and caspase-3/7 activity, along with LDH release, were determined in the co-culture after exposure to F837/76 (Nhe+Hbl) supernatant at sublethal and lethal doses. As shown in Figure 9(A), caspase-1 activity exhibited a modest increase at the lethal dose (~1.5-fold vs the negative control), while remaining unchanged at the sublethal dose, with no significant differences relative to the control or between toxin concentrations. Caspase-3/7 activity showed a dose-dependent increase, reaching approx. 1.6-fold at the lethal dose and displaying a significant increase relative to untreated cells, while for the sublethal dose no significant difference was detected from the control (Figure 9(B)). LDH release was significantly elevated at both toxin doses (sublethal: ~1.6-fold; lethal: ~1.8-fold), in accordance with the loss of membrane integrity, with no significant difference observed between sublethal and lethal exposure (Figure 9(C)). In summary, the Nhe+Hbl containing supernatant induced a significant increase in caspase-3/7 activity (apoptosis) as well as LDH release (loss of membrane integrity), while caspase-1 activity (pyroptosis) increased non-significantly.

## Discussion

Although the tripartite enterotoxins Nhe and Hbl are well-characterized as the main drivers of the diarrheal disease caused by *B. cereus*, detailed information on how human intestinal epithelia respond to enterotoxin exposure over time is limited. To address this gap, we used a polarized Caco-2/HT29-MTX 9:1 co-culture to determine cell binding, membrane stability, cell viability, transcriptional response and the induction of cellular pathways after enterotoxin exposure.

### Comparable binding, but cell line-specific cytotoxic activity of the enterotoxins

The first step of pore assembly is interaction of the binding component, Hbl B [8,9] or NheB-C [12,53], with the target cells. Using rHbl B as an example, the present study demonstrated a similar binding capacity to Caco-2 and HT29-MTX cells, which might be a first hint that both cell lines are similarly equipped with Hbl-specific receptors LITAF and/or CDIP1 [16]. LITAF exists on the surface of numerous human cell types, tissues, and organs and is further involved in the activation of inflammation [19]. Thus, it is assumed that *B. cereus* might trigger cell‑death responses in the host by specifically targeting this receptor via Hbl [19]. Additionally, immunogold transmission electron microscopy localized rHbl B at the cell membranes of Caco-2 and HT29-MTX, particularly in the microvilli region. Although not yet quantified, we observed enhanced repulsion of these cell structures and increased cellular debris in the extracellular space, which could suggest a defense mechanism used by the cells to dispose of the external protein. Similarly, we observed in an older, not yet published experiment, changes in the surface structure of Vero cells after exposure to partially neutralized rHbl (L2+L1+B). Membrane disruption is shown (Supplementary Fig. S8A and B), similar to observations made using liposomes [20,21]. We further found an increased appearance of membrane vesicles (Supplementary Fig. S8C), which points to the onset of cellular repair mechanisms such as blebbing and shedding [86]. Blebbing is a widespread cellular response to plasma membrane disruption based on calcium-dependent actomyosin contraction. It results from impaired interaction between the plasma membrane and the cytoskeleton. Key players are lipid domains in the plasma membrane reacting to toxin attachment even before membrane disruption. This could be one of the reasons why we see a cellular reaction to only rHbl B, which is not capable of pore formation without the components L1 and L2 [32]. Blebbing then allows the shedding of microvesicles, one of the major plasma membrane repair mechanisms [53]. Similarly, after *Serratia marcescens* hemolysin exposure, thinning of both insect and mammalian gut epithelium was observed, which recovered to original thickness in only a few hours [54]. During this process, enterocytes do not lyse but extrude large parts of their apical cytoplasm including affected organelles, thus expelling defective compounds and possible invading bacteria. The rapid recovery involves CyclinJ-dependent signaling between enterocytes [54]. Also, the administration of *B. cereus* culture supernatants (whole toxins) rapidly induced microvilli effacement, pronounced apical surface alterations, and epithelial cell damage [55,56]. Consistently, surface roughening/inflammatory-like changes, nuclear and cytoplasmic injury, and membrane perforation and organelle damage following *B. cereus* exposure were reported [57].

In contrast to comparable rHbl B binding, WST-1-based reciprocal IC_50_ profiling (Figure 2) ranked Caco-2 cells as most sensitive toward Hbl or Nhe+Hbl, HT29-MTX cells as least sensitive, and the co-culture as intermediate, consistent with a reduction of effective toxin contact with the plasma membrane by mucus and goblet-like features. Variations in the susceptibility of different cell lines toward Hbl or Nhe have already been demonstrated, with Caco-2 showing equal sensitivity to both enterotoxin complexes, while HT29 or HT29-MTX were not included in this comparative analysis [33]. Notably, against the co-culture, NVH 0075–95 (Nhe) appeared more cytotoxic than F837/76 (Nhe+Hbl). Although uniform NheB and Hbl L2 production of all strains applied in this study was assured via previously performed enzyme immuno assays, deviations of the other subunits and thus their concentration ratio might be possible, albeit unlikely, which can have an effect on cytotoxic activity [8,10]. Moreover, the toxic effect of rHbl would seem to be lower than that of the applied *B. cereus* culture supernatants. This could be due to genetic modifications, such as the insertion of a linker peptide and a Strep tag for purification [32], due to protein overexpression in *E. coli* [32], or due to differences in applied concentrations. The enterotoxin concentrations in the culture supernatants are not clearly determinable in the above‑mentioned enzyme immuno assays. However, as rHbl B was used as example in all binding experiments, it was included in all cytotoxicity tests as well. Further possibly secreted virulence factors contributing to total cytotoxicity of the applied supernatants can be largely excluded, as earlier studies revealed no remaining cytotoxic activity after removal of Nhe and Hbl via immunoaffinity chromatography [33]. Comparing differentiation states confirmed that barrier maturity strongly affects cell susceptibility (Figure 2(C)). 24 h co-cultures were considerably more sensitive than 28 d differentiated co-cultures, consistent with enhanced polarization and tighter junctional organization as well as mucus production in mature monolayers [28,30,58,59].

### Interference of the enterotoxins with membrane stability and ion transport

To interpret barrier and transport readouts under standardized conditions, the polarized Caco-2/HT29-MTX co-culture was exposed to *B. cereus* enterotoxins after TEER had reached a stable plateau. Time- and toxin-dependent decline in TEER was observed (Nhe+Hbl > Nhe >Hbl >rHbl), indicating compromised barrier function by direct membrane permeabilization, and paracellular leakage. Further, basolateral exposure showed a slightly stronger effect compared to apical toxin treatment (compare Figure 3). This may reflect polarity-dependent differences in toxin access or differences in susceptibility due to epithelial organization and apical surface properties. Although the apical mucus layer is described as the first barrier that prevents bacteria and presumably their toxins from reaching the epithelium [28,59], the present data do not directly prove a mucus-specific protective effect. In an earlier study, Castiaux et al. concluded that the mucus secreted by HT29-MTX cells did not form a significant protective barrier for Caco-2 cells against *B. cereus* culture supernatants under the conditions tested [60]. In case of a *B. cereus* toxico-infection in real life, the enterotoxins would almost exclusively attack from the apical region. Basolateral exposure, however, is possible in case of a systemic infection and enterotoxin attack from the bloodstream or intercellular space.

Membrane lesions caused by pore-forming toxins can directly disturb ion gradients. The toxin-containing supernatants used in the present study, especially F837/76 (Nhe+Hbl), increased short-circuit current (Isc), while epithelial resistance (Rt) steadily diminished over time (see Figure 4 and Supplementary Figures S2 and S3). As Isc integrates net electrogenic ion transport across the epithelium, this increase is understood as pro-secretory shift often driven by anion secretion, which might induce osmotic water migration into the lumen [61,62]. The Hbl pores are characterized as small (approx. 1.2 nm channel diameter), moderately cation selective, unstable and of limited lifespan [15,63]. NheB-C complexes assemble pre-pores with approx. 2 nm channel diameter [11,12], while the complete pore is described as cation selective and very stable with a channel diameter > 2.8 nm [13,14]. Nhe-induced Ca^2+^ influx into Vero cells has been described [18], as well as K^+^ efflux triggered by both Hbl and Nhe pores [20,21]. Among the conditions tested in the present study, strain F837/76 (Nhe+Hbl) produced the most pronounced, statistically significant, yet gradual Rt reduction. Consistent with its strong Isc response, this indicates a real electrogenic ion transport response rather than primary junctional disruption [62]. In contrast, Rt did not decrease significantly with the Nhe-only or Hbl-only supernatants under the tested conditions. In parallel, a reduction in Rt is widely used as an indicator of impaired barrier integrity and increased permeability, consistent with paracellular shunting or toxin-induced epithelial damage. Furthermore, the combination pattern of increasing Isc and decreasing Rt supports a mixed diarrheal phenotype, in which dysregulated epithelial transport corresponds with barrier leakage rather than representing a pure secretory response [64]. Notably, the Rt decline in the Ussing setup was slower than the pronounced TEER decrease measured with the EVOM^2^ system, underscoring that “resistance” readouts are method- and context-dependent and can reflect a combination of paracellular (junctional) permeability and transcellular conductance/ion-channel activity rather than tight junctions alone [36]. Consequently, a decrease in resistance should not be interpreted as direct evidence of complete loss of epithelial viability, because TEER/Rt can change without a proportional cytotoxicity signal and its time course can capture distinct stages of barrier perturbation [65]. Overall, the convergence of multiple readouts (TEER, WST-1, Ussing chamber measurements, and EM) strengthens the conclusion that also in the polarized Caco-2/HT29-MTX co-culture, which mimics the human intestinal epithelium more closely than other models, *B. cereus* enterotoxin-dependent barrier impairment leads to cell membrane and surface damage and thus epithelial dysfunction underlying diarrhea.

### Induction of inflammation- and stress-associated pathways

Via RNA sequencing, a time- and dose-dependent transcriptional response of the polarized Caco-2/HT29-MTX co-culture to enterotoxin exposure (Nhe+Hbl) was detected in the present study. Previously determined lethal (IC_90_) and sublethal (IC_10_) toxin doses were applied for 2 h. Under lethal exposure, the key signal pathways and responses were already triggered. Additionally, the 8 h application was carried along simulating long-term enterotoxin exposure as “worst case scenario,” for example when a *B. cereus* strain is both strongly adherent and toxin-producing [31]. While inhibition of cell proliferative signaling (lethal) or cell metabolism (sublethal) took place after 2 h, several inflammation- and stress-associated genes such as *JUN* and *FOS* (Table 1) and pathways including *ATF3* and p53-associated stress/DNA-damage programs together with prominent TNFα/NF-κB and IL6–JAK–STAT3–linked inflammatory modules (Figure 7) were enriched. Shifts and enrichments over time pointed out progressive stress accumulation and ongoing remodeling (Figure 7 and 8). This combination is typical for epithelial sensing of membrane and metabolic stress, where ion disequilibrium and energetic stress drive MAPK/ISR signaling while danger responses enhance NF-κB-dependent inflammation [53,66]. In accordance with this, the pore-forming toxin alveolysin from *B. cereus* was shown to disrupt tight and adherens junctions via CD59-dependent PI3K/AKT signaling and downstream cytoskeletal changes, providing a mechanistic link between exposure to pore forming toxins and barrier failure beyond just “hole formation” [67]. An earlier study already showed that Nhe-induced Ca^2+^ influx led to activation of apoptosis signal-regulating kinase 1 (ASK1) and p38 MAPK in Vero cells. This was followed by caspase-8 and caspase-3 activation which induced apoptosis [18]. Further, Hbl- [21] as well as Nhe- [20] induced K^+^ efflux triggered mortality of macrophages via the NLRP3 inflammasome. This NOD-, LRR- and pyrin domain-containing protein 3 is the sensor protein in the inflammasome complex with apoptosis-associated-speck-like adaptor protein and cysteine protease caspase-1. Subsequent secretion of the proinflammatory cytokines interleukin-1β and interleukin-18 ultimately led to pyroptosis [19–21]. Regarding the response of human intestinal cells toward *B. cereus* infection, a “more energetic” phenotype was observed due to enhanced flux through oxidative phosphorylation and glycolysis and higher ATP levels [68]. Notably, long-time exposure showed a larger number of down-regulated genes at lethal compared to sublethal doses, e.g. *SLC2A1* and *ANKRD1* (Figure 6 and Supplementary Table S3), and a relative downshift of inflammatory/tight-junction hallmarks (Figure 8). These results may reflect two non-exclusive processes: First, cells proceed toward advanced injury/apoptosis, where global mRNA decay and its influence on transcription can make down-regulation more apparent in bulk RNA sequencing, or second, a surviving subpopulation shows metabolic rewiring consistent with adaptation/energy management rather than strong early inflammatory response [69,70]. In this context, it should be mentioned that the initial quality check of our RNA showed degradation after 8 h of lethal exposure, but that it nonetheless remained of sufficient quality for the subsequent sequencing reactions (Azenta Life Sciences). Moreover, we tested if the analysis tool chosen has a decisive influence on the results and conducted two analyses. KEGG pathway enrichment analysis independently confirmed the primary Hallmark pathways identified with pre-ranked Gene Set Enrichment Analysis (GSEA) (Supplementary Fig. S6 and S7).

The identified transcriptome patterns were mirrored by cell-death markers, which suggest a progression from regulated death signaling to terminal membrane failure as exposure intensifies. Executioner caspase-3/7 activation supported engagement of apoptotic machinery, whereas increased LDH release indicated loss of membrane integrity/lysis, often interpreted as late-stage cytotoxicity or secondary necrosis following severe injury [71]. By contrast, caspase-1 activity showed a non-significant increase under the conditions chosen, arguing against inflammasome-driven pyroptosis as the dominant terminal pathway in our epithelial co-culture, even though inflammatory transcription was strongly induced. This noticeable difference might biologically make sense: Nhe and Hbl can activate NLRP3 inflammasome signaling and pyroptosis in relevant immune contexts, and pore-forming toxins in general can trigger NLRP3 via K^+^ efflux. Nevertheless, the epithelial monolayers may not execute pyroptosis efficiently under the conditions tested, due to differences in priming, inflammasome competence, or downstream gasdermin activation, resulting instead in an inflammatory stress response that culminates primarily in apoptosis at higher toxin burden. However, these observations require further downstream investigations to be verified. It should also be noted that baseline signals for caspase-1, caspase-3/7, and LDH were elevated relative to background, consistent with spontaneous cell turnover and low-level apoptosis as well as basal (“spontaneous”) LDH release that can occur in long-time differentiated epithelial cultures with reduced proliferative activity [72].

### Evidence for cell recovery

Epithelial cells can mitigate pore-forming toxin injury through multiple membrane-repair pathways, including patch formation, endocytic removal of toxin lesions, clogging of the pores, and shedding of damaged membrane segments via blebbing [53,73]. During bacterial infections, such as the *B. cereus* toxico-infection, the faith of infected cells depends on the coordinated interplay of such cell regeneration processes [53]. This can result in epithelial thinning and subsequent recovery, which is designated as fast and efficient response to intestinal infections, while at this the pore-forming toxins are understood as alarm signals [54]. In line with the concept of reversible, sublethal poration, *B. cereus* hemolysin II was reported to permit trypan blue entry into viable cells, indicating transient membrane permeabilization and recovery after toxin removal under non-lethal conditions [74]. Consistent with this, we observed partial and toxin-dependent restoration of viability of the (non-differentiated) Caco-2/HT29-MTX co-culture as reflected by reciprocal IC_50_ values (Figure 5(A)) and live-cell imaging after exposure to sublethal toxin doses (Supplementary recordings S7-9). Moreover, the barrier integrity of the differentiated co-cultures could be restored (Figure 5(B). Notably, this seemed to occur in a significantly shorter time than the formation of the original barrier (Figure 3(A)), possibly due to the use of low (sublethal) toxin concentrations or the activation of repair mechanisms. The fact that specific repair mechanisms were involved rather than proliferation of randomly non-infected cells can be inferred from the obtained transcriptome data. GSEA (Figure 7) revealed activation of epithelial remodeling and stress-adaptation programs, such as TGF-β signaling, which is important for epithelial remodeling and intestinal mucosal healing [75,76]. Moreover, the detected IL6–JAK–STAT3 pathway is involved in intestinal epithelial proliferation and repair after injury [77] and WNT/β-catenin, Notch and Hedgehog pathways are typically associated with intestinal epithelial homeostasis, stem cell niche, regeneration, and differentiation [78,79]. The detected PI3K-AKT-mTOR/mTORC1 pathway is also important for intestinal epithelial repair and generally in epithelial wound healing [80,81]. These findings reflect repair priming in surviving epithelial cells. Gradual reattachment, spreading, regrowth and reconstruction of barrier integrity support the reversibility of the effect of the pore-forming enterotoxins of *B. cereus*, depending on exposure magnitude and duration [82,83].

### Limitations of this study and perspective on forthcoming investigations

With the application of the Caco-2/HT29-MTX co-culture, the present study focused on a more complex target system for the *B. cereus* enterotoxins, rather than on single cell lines. Nevertheless, determining gene expression and the onset of signaling pathways in Caco-2, HT29-MTX and further intestinal cell types in a comparative manner would provide further insights into the onset of the diarrheal disease caused by *B. cereus*. Further, it has not yet been fully determined which intestinal cell types are most sensitive and thus, the primary target of the *B. cereus* enterotoxins. The gained transcriptome data point toward activation of stress-adaptation and repair-associated pathways in the Caco-2/HT29-MTX model. These findings reflect repair priming in surviving epithelial cells, but we have not yet demonstrated a direct involvement of these pathways as well as the molecular process of cellular repair. Thus, the link between enhanced expression of these pathways and the restoration of cellular and barrier integrity has to be experimentally proven in future studies. The same applies to the possible mutual balancing of repair mechanisms and apoptotic signaling pathways.

*B. cereus*-induced diarrhea is often acute and self-limiting. Rapid epithelial stress response and recovery could help alleviate the symptoms after toxin exposure is over. However, this remains a hypothesis as our model does not include immune cells, the intestinal microbiome, the stem cell niche, or true tissue regeneration yet. Nevertheless, with the introduction of HT29-MTX cells, we started integrating the role of mucus into our model. In an earlier study, we already demonstrated multiple interactions between *B. cereus* and mucin, such as directed movement toward mucin, triggered spore germination, strain-specific mucin degradation, enhanced growth, enhanced enterotoxin production, major transcriptional changes upon mucin contact, and protection of the enterotoxins from enzymatic degradation [84]. So far, protection of Caco-2 cells from *B. cereus* enterotoxins by HT29-MTX-secreted mucus could not be confirmed [60], but it is suggested that mutual communication between epithelial cells and the immune system is meditated via mucins [59]. This, as well as the protective effects of the mucus layer on Caco-2 cells under the conditions applied in our experiments, still need to be elucidated. To investigate this and even more complex target structures of the *B. cereus* enterotoxins, the application of intestinal organoids would constitute a next step.

## Conclusions

The present study describes the Caco-2/HT29-MTX 9:1 co-culture as a suitable model for investigating the impact of *B. cereus* enterotoxins in a simulation more closely resembling the human intestine. Enterotoxin-containing supernatants induced a time-dependent barrier breakdown (decline of TEER/Rt) and altered epithelial ion transport (increase of Isc), with the dual-toxin (Nhe+Hbl) producing strain F837/76 causing the strongest effects. Cell type- and enterotoxin-specific differences were also evident in terms of cell viability. Total transcriptome analyses of the differentiated co-culture revealed a dose- and time-dependent cell-response program dominated by TNFα/NF-κB and MAPK stress signaling and p53/apoptosis pathways, with later signatures of metabolic remodeling. Cell-death assays supported this, showing increased caspase-3/7 and LDH as well as minimal caspase-1 changes, consistent with early inflammatory response, apoptotic cell death and membrane damage at late exposure time as the main outcomes. Nevertheless, cell recovery and restoration of the epithelial barrier integrity were also observed at low toxin concentrations, which corresponds to the detected repair priming in surviving epithelial cells.

## Supplementary Material

Supplement Revision 24_06_26 unmarked.docx

## Data Availability

RNA sequencing data have been deposited in the NCBI GEO database (https://www.ncbi.nlm.nih.gov/geo/) under accession number GSE293991 [85]. All other raw data that support the findings of this study as well as the Supplementary recordings S1-9 are deposited at https://figshare.com and can be accessed via https://doi.org/10.6084/m9.figshare.31537750 [87].

## Open scholarship

This article has earned the Center for Open Science badge for Open Data. The data are openly accessible at https://doi.org/10.1080/23311908.2023.2245607
