# Supplementary material for: Bacillus cereus enterotoxins change epithelial barrier function and ion transport in a Caco-2/HT29-MTX co-culture model, provoking inflammation, apoptosis and membrane damage
Source: Virulence. 2026 Aug 27;17(1):2725391. doi: 10.1080/21505594.2026.2725391 (PMC13556967; doi:10.1080/21505594.2026.2725391)
Supplement: Supplementary file 1 [file KVIR_A_2725391_SM8758.docx]

***Bacillus cereus* enterotoxins change epithelial barrier function and ion transport in a Caco-2/HT29-MTX co-culture model, provoking inflammation, apoptosis and membrane damage**

**Manar Albahri, Bettina Seeger, Andreas Beineke, Tuba Esatbeyoglu, Madeleine Plötz, Nadja Jessberger**

**Supplementary material**

**Table S1**. List of primers and their properties used in qRT-PCR analyses to validate RNA sequencing results.

| **Gene** | **Primer** | **Sequence (5’-3’)** | **Fragment length [bp]** | **Primer efficiency [%]** | **Reference** |
| --- | --- | --- | --- | --- | --- |
| Reference genes | | | | | |
| *YWHAZ* | fw | GCCAGCAGAACATCCAGTCAT | 196 | 89 | NCBI |
|  | rev | CCAAGATGACCTACGGGCTC |  |  |  |
| *RPLP0* | fw | ACGGGTACAAACGAGTCCTG | 75 | 83 | NCBI |
|  | rev | GGCCTTGACCTTTTCAGCAAG |  |  |  |
| Up-regulated | | | | | |
| *JUN* | fw | GTGCCGAAAAAGGAAGCTGG | 175 | 99 | NCBI |
|  | rev | CCACTCTACGATCAGGGTGGC |  |  |  |
| *DUSP1* | fw | CCACTCTACGATCAGGGTGGC | 136 | 108 | NCBI |
|  | rev | TGGGACAATTGGCTGAGACG |  |  |  |
| *CXCL2* | fw | GAAAGCTTGTCTCAACCCCG | 82 | 104 | NCBI |
|  | rev | TGGTCAGTTGGATTTGCCATTTT |  |  |  |
| *FOS* | fw | GATACACTCCAAGCGGAGACA | 72 | 98.1 | NCBI |
|  | rev | CAGGTTGGCAATCTCGGTCT |  |  |  |
| *TSC22D2* | fw | AGAACAAGCAATGGATCTGGT | 199 | 101 | NCBI |
|  | rev | AGAAGTGCTACCAGGATTGGC |  |  |  |
| *TNFAIP3* | fw | CTGAAAACGAACGGTGACGG | 114 | 101 | NCBI |
|  | rev | TTCCTTGAGCGTGCTGAACA |  |  |  |
| *MAAF* | fw | GAGGGCACCTTCTGCAAACA | 73 | 111 | NCBI |
|  | rev | GCTCAGCTCTCGCTTGATCTTT |  |  |  |
| Down-regulated | | | | | |
| *LYZ* | fw | AGCCGCTACTGGTGTAATGA | 155 | 100 | NCBI |
|  | rev | CATGCCACCCATGCTCTAAT |  |  |  |
| *ST3GAL3* | fw | ATCTTCCCCCGGTTCTCCAA | 79 | 85.8 | NCBI |
|  | rev | CGAACTCCCGGATTCTAGCC |  |  |  |
| *TRPV1* | fw | CATCGACACCTGCGAGAAGA | 78 | 96.6 | NCBI |
|  | rev | ATGTCGTGGCGATTAGGGGT |  |  |  |

**Table S2.** Top 30 differentially expressed genes (DEGs) under enterotoxin exposure compared to non-treated cells, sorted by their adjusted p-value. The Wald test was used to generate p-values and log_2_ fold changes (FC). Genes with an adjusted p-value < 0.05 and absolute log_2_ fold change (FC) > 1 were designated differentially expressed. p-values were adjusted for multiple testing using the Benjamini–Hochberg procedure.

| **neg vs 8h-sub** | | **neg vs 8h-let** | | **neg vs 2h-sub** | | **neg vs 2h-let** | |
| --- | --- | --- | --- | --- | --- | --- | --- |
| **Gene name** | **log_2_FC** | **Gene name** | **log_2_FC** | **Gene name** | **log_2_FC** | **Gene name** | **log_2_FC** |
| *SLC38A2* | 3.59 | *RCAN1* | 3.1 | *EGR1* | 2.03 | *JUN* | 4.74 |
| *ANKRD1* | 4.72 | *EGR1* | 3.67 | *RCAN1* | 1.13 | *CXCL2* | 4.42 |
| *TRIB1* | 3.6 | *TNFAIP3* | 3.79 | *FOS* | 1.69 | *RCAN1* | 3.86 |
| *GEM* | 4.52 | *BMP2* | 3.83 | *NR4A1* | 1.26 | *NFKBIA* | 3.52 |
| *SLC20A1* | 3.62 | *MT-TH* | 3.97 | *IER3* | 1.41 | *TNFAIP3* | 2.95 |
| *EPHA2* | 3.46 | *PPP1R15A* | 4.06 | *JUN* | 1.15 | *FOS* | 4.32 |
| *PPP1R15A* | 3.07 | *JUNB* | 4.07 | *FOSB* | 1.41 | *PPP1R15A* | 2.66 |
| *MAFF* | 4.12 | *CYR61* | 4.17 | *CYR61* | 1.17 | *ATF3* | 3.28 |
| *JUNB* | 3.39 | *HSPA1A* | 4.22 | *ATF3* | 1.03 | *DUSP1* | 3.63 |
| *BMP2* | 3.77 | *HSPA1B* | 4.26 | *GDF15* | 1.00 | *JUNB* | 3.01 |
| *AREG* | 3.16 | *ATF3* | 4.36 | *ANKRD1* | 1.12 | *EGR1* | 2.81 |
| *IER3* | 3.35 | *NFKBIA* | 4.44 | *CCL2* | 2.9 | *CXCL3* | 5.11 |
| *TM4SF1* | 3.18 | *GEM* | 4.55 | *CXCL2* | 1.02 | *NR4A1* | 2.94 |
| *CYR61* | 3.63 | *DUSP1* | 4.76 | *PTGS2* | 1.22 | *MAFF* | 3.12 |
| *GPRC5A* | 3.47 | *MT-TG* | 4.91 | *AL161431.1* | 1.85 | *ELF3* | 2.30 |
| *BHLHE40* | 2.93 | *CXCL2* | 5.04 | *EGR3* | 1.9 | *GEM* | 3.27 |
| *SLC2A1* | 2.96 | *FOS* | 5.62 | *NR4A3* | 1.9 | *ZBTB10* | 2.73 |
| *KLF6* | 3.01 | *JUN* | 6.35 | *NFKBID* | 1.09 | *SLC38A2* | 2.19 |
| *EHD4* | 2.71 | *MT-ND6* | 3.42 | *CCL20* | 1.2 | *ZFP36* | 2.19 |
| *DKK1* | 4.28 | *ZBTB10* | 3.59 | *KLK11* | -3.52 | *BMP2* | 2.72 |
| *DUSP1* | 3.77 | *MAFF* | 4.1 | *AC017002.1* | 1.63 | *SLC25A25* | 2.42 |
| *NFKBIA* | 2.98 | *TSC22D2* | 2.84 | *CXCL3* | 1.32 | *C6orf222* | 2.15 |
| *SLC2A3* | 3.20 | *SLC38A2* | 2.72 | *CAPN9* | -2.67 | *CXCL1* | 3.78 |
| *JUN* | 3.07 | *IER5* | 3.48 | *REG4* | -3.28 | *IER3* | 2.65 |
| *SLC5A3* | 2.75 | *MT-ND5* | 3.32 | *TACSTD2* | -2.22 | *NFKBID* | 3.67 |
| *TNFAIP3* | 2.72 | *FOSB* | 4.34 | *CCDC9B* | -1.59 | *TSC22D2* | 1.8 |
| *LDLR* | 2.39 | *MT-TL2* | 3.94 | *RAB27B* | -3.50 | *NFKB2* | 2.32 |
| *FOS* | 3.58 | *TRIB1* | 3.19 | *ABLIM3* | -2.58 | *FOSB* | 2.95 |
| *SLC25A25* | 3.04 | *DNAJB1* | 3.21 | *EGR2* | 1.02 | *EGR2* | 3.39 |
| *EGR1* | 3.01 | *MT-CO1* | 3.21 | *VSIG2* | -1.93 | *CCL20* | 3.47 |

**Table S3.** Top 30 differentially expressed genes (DEGs) for pairwise comparison between enterotoxin exposure conditions, sorted by their adjusted p-value. The Wald test was used to generate p-values and log_2_ fold change s(FC). Genes with an adjusted p-value < 0.05 and absolute log_2_ fold change (FC) > 1 were called as differentially expressed genes. p-values were adjusted for multiple testing using the Benjamini–Hochberg procedure.

| **8h-sub vs 8h-let** | | **2h-sub vs 8h-sub** | | **2h-sub vs 2h-let** | | **2h-let vs 8h-let** | |
| --- | --- | --- | --- | --- | --- | --- | --- |
| **Gene name** | **log_2_FC** | **Gene name** | **log_2_FC** | **Gene name** | **log_2_FC** | **Gene name** | **log_2_FC** |
| *SLC2A1* | -2.54 | *GEM* | 3.85 | *JUN* | 3.57 | *MT-TC* | 2.77 |
| *HSPA1B* | 2.81 | *GPRC5A* | 3.22 | *RCAN1* | 2.71 | *MT-TL2* | 3.59 |
| *MT-TC* | 2.96 | *SLC2A1* | 2.94 | *CXCL2* | 3.38 | *MT-TG* | 4.29 |
| *HSPA1A* | 3.00 | *SLC38A2* | 3.07 | *NFKBIA* | 2.84 | *MT-TH* | 3.22 |
| *JUN* | 3.32 | *SLC20A1* | 3.24 | *TNFAIP3* | 2.33 | *RPS23* | 2.14 |
| *MT-TL2* | 3.83 | *TRIB1* | 3.00 | *ELF3* | 1.85 | *MTATP6P1* | 2.72 |
| *MT-TH* | 3.95 | *SLC2A3* | 3.05 | *CXCL3* | 3.77 | *DKK1* | 3.43 |
| *MT-TG* | 4.91 | *MAFF* | 3.16 | *ZBTB10* | 2.31 | *MT-ND6* | 3.00 |
| *HKDC1* | -2.70 | *BMP2* | 3.22 | *DUSP1* | 2.89 | *EDN1* | 2.98 |
| *ANKRD1* | -3.45 | *AC016831.1* | 3.49 | *PPP1R15A* | 2.02 | *CITED2* | 2.40 |
| *NEAT1* | -2.05 | *EPHA2* | 2.88 | *ATF3* | 2.22 | *MT-TK* | 3.71 |
| *CXCL2* | 2.75 | *KLF6* | 2.41 | *FOS* | 2.62 | *MT-ND4L* | 2.29 |
| *CBX5* | 2.43 | *EHD4* | 2.42 | *TSC22D2* | 1.65 | *CBX5* | 1.98 |
| *MTATP6P1* | 2.87 | *PPP1R15A* | 2.42 | *MAFF* | 2.16 | *MT-ND3* | 2.31 |
| *MT-TK* | 3.92 | *MRPS6* | 2.56 | *JUNB* | 2.26 | *CYR61* | 2.39 |
| *LAMC2* | -2.12 | *TM4SF1* | 2.92 | *GEM* | 2.60 | *TRIB1* | 1.89 |
| *FOS* | 2.08 | *JUNB* | 2.63 | *BMP2* | 2.18 | *RGS2* | 2.61 |
| *RPS23* | 2.00 | *TXNRD1* | 2.44 | *CXCL1* | 3.46 | *TM4SF1* | 1.90 |
| *FLNC* | -2.16 | *TNFRSF12A* | 2.41 | *SLC25A25* | 1.89 | *H3F3B* | 1.57 |
| *MICAL2* | -2.45 | *SLC5A3* | 2.59 | *HSPA1A* | 2.49 | *MT-TY* | 2.23 |
| *FLNA* | -1.88 | *ZBTB7A* | 2.33 | *ZFP36* | 1.68 | *RPL13A* | 1.82 |
| *GPRC5A* | -1.86 | *FLNC* | 2.26 | *NR4A1* | 1.66 | *IER5* | 1.82 |
| *SLC2A3* | -1.96 | *HKDC1* | 1.93 | *NFKBID* | 2.56 | *FTH1* | 1.59 |
| *SPTBN1* | -1.85 | *TINAGL1* | 2.42 | *C6orf222* | 1.56 | *PPDPF* | 1.77 |
| *FOSB* | 2.55 | *ERRFI1* | 2.91 | *NFKB2* | 1.83 | *ERRFI1* | 2.17 |
| *AC016831.1* | -3.17 | *SLCO4A1* | 2.97 | *JUND* | 1.53 | *PPP1R15A* | 1.38 |
| *AKR1C1* | -1.8 | *HK2* | 2.85 | *IFRD1* | 1.52 | *MTCO1P12* | 2.61 |
| *ITGA2* | -2.5 | *TNFRSF10B* | 1.93 | *SLC38A2* | 1.68 | *RPL36* | 1.50 |
| *PRSS22* | -2.99 | *PFKFB3* | 2.57 | *CSRNP1* | 1.83 | *KLF4* | 1.88 |
| *EIF4EBP2* | 1.70 | *EPOP* | 2.87 | *PPP1R10* | 1.41 | *MT-TW* | 2.53 |

**Figure S1.** Comparison of enterotoxin production between the applied *B. cereus* reference strains. Reciprocal titres were determined via sandwich enzyme immuno assays, using the monoclonal antibodies 1A12 and 1H9-HRP for Hbl L2 [1] as well as 2B11 and 1E11-HRP for NheB [2] detection. The quantities of Hbl L2 and NheB cannot be compared on the basis of the determined titres, as these depend on the affinity of the respective antibodies.

**Figure S2.** Comparison of the short-circuit currents (Isc) in Caco-2/HT29-MTX co-cultures after 2.5 h of toxin exposure. *B. cereus* enterotoxin-containing supernatants were added to the apical side of the Ussing chamber at 1:40 dilution. Bacterial culture medium CGY or rHbl elution buffer was applied as corresponding negative control. The chamber was maintained at 37 °C using a water bath, and carbogen was continuously supplied to homogenize the buffer and support cell viability. Isc before treatment was defined as the mean of 10 measurements immediately before toxin addition, and Isc after treatment as the highest value recorded after 2.5 h exposure. P-values indicate statistically significant differences in Isc values before and after treatment within each condition. Significance was assessed using repeated-measures ANOVA followed by the Holm-Sidak multiple-comparisons test. ns: not significant; *: p < 0.05; **: p < 0.01.

**Figure S3.** Changes in transepithelial resistance (Rt) in Caco-2/HT29-MTX co-cultures after 2.5 h of toxin exposure. *B. cereus* enterotoxin-containing supernatants were added to the apical side of the Ussing chamber at 1:40 dilution. Bacterial culture medium CGY or rHbl elution buffer was applied as corresponding negative control. The chamber was maintained at 37 °C using a water bath, and carbogen was continuously supplied to homogenize the buffer and support cell viability. Baseline Rt (before treatment) was defined as the mean of 10 measurements immediately before toxin addition, and Rt after treatment as the minimum value recorded during the 2.5 h exposure period. P-values indicate statistically significant differences in Rt values before and after treatment within each condition. Significance across biological replicates was assessed using repeated-measures ANOVA followed by the Holm-Sidak multiple-comparisons test. ns: not significant; *: p < 0.05.

**Figure S4.** Global transcriptional changes across compared conditions in Caco-2/HT29-MTX co-cultures after exposure to the supernatant of *B. cereus* strain F837/76 (Nhe+Hbl), visualized by volcano plots. Each dot represents a single gene, with log₂ fold change on the x-axis and -log₁₀ of its adjusted p-value on the y-axis. Genes with an adjusted p-value < 0.05 and log₂ fold change > 1 are highlighted in red (up-regulated), genes with an adjusted p-value < 0.05 and log₂ fold change < -1 are highlighted in blue (down-regulated). neg. ctrl: untreated co-culture, let: lethal dose (IC_90_), sub: sublethal dose (IC_10_).

**Figure S5.** Examples of GSEA enrichment plots illustrating both positively and negatively enriched Hallmark gene sets in the co-culture model after 2 h toxin exposure at a sublethal dose. Hallmark TNFα-signalling via NF-κB was positively enriched (left diagram; NES = 3.15; FDR q-value = 0), Hallmark oxidative phosphorylation was negatively enriched (right diagram; NES = -2.07; FDR q-value = 0). The plots display the running enrichment score (ES) across the rank-ordered gene list, and the locations of gene set members, highlighting where the respective signatures are concentrated along the expression-based ranking.

**Figure S6**. KEGG pathway enrichment analysis of significantly DEGs in cells exposed to supernatant of strain F837/76 (Nhe+Hbl) compared to untreated cells (neg. ctrl). For each comparison, the top 20 enriched pathways (FDR cut off = 0.05) were selected based on -log_10_ (FDR) value. Analysis and visualisation were conducted using ShinyGO version 0.85.1 [3]. Bar lengths indicate the number of genes associated with each pathway, while colour represents the -log_10_ (FDR). FDR (false discovery rate) controls the expected proportion of false positives among the declared significant results. **A.** 2 h lethal dose. Predominantly enriched KEGG pathways were associated with cytokine and inflammation-related signalling, including the IL-17, TNFα, NF-κB, and chemokine signalling pathway as well as cytokine-cytokine receptor interaction. **B.** 2 h sublethal dose. **C.** 8 h lethal dose. Considerable enrichment of the MAPK signalling pathway, oxidative phosphorylation, autophagy and mitophagy, cellular senescence, and the p53 signalling pathway. **D.** 8 h sublethal dose. Enrichment transitioned towards metabolic and alongside signalling pathways such as the MAPK, PI3K-Akt, and Hippo signalling pathways, in addition to stress and remodelling pathways associated with long-term adaptation, including cellular senescence, efferocytosis, and mitophagy.

**Figure S7.** KEGG pathway enrichment analysis of significantly DEGs across exposure times (2 h vs. 8 h) and toxin doses (lethal vs. sublethal) for cells treated with supernatant of strain F837/76 (Nhe+Hbl). Pairwise comparisons include **A.** dose effect at 2 h. Demonstrated were cell-fate regulation and inflammatory signalling, including the cell cycle, necroptosis, TNFα signalling, NF-κB signalling, and IL-17 signalling. These support a more pronounced early inflammatory and cell death-related response under lethal conditions. **B.** time effect under lethal exposure, **C.** time effect under sublethal exposure, and **D.** dose effect at 8 h. For each comparison, the top 20 enriched pathways (FDR cut off = 0.05) were selected based on -log_10_ (FDR) value. Analysis and visualisation were conducted using ShinyGO version 0.85.1 [3]. Bar lengths indicate the number of genes associated with each pathway, while colour represents the -log_10_ (FDR). FDR (false discovery rate) controls the expected proportion of false positives among the declared significant results.

**Figure S8.** Immunogold-localization of rHbl (L2+L1+B) on the surface of Vero cells via electron microscopy. Hbl L2-specific and toxin-neutralizing mAb 1H9 [1] as well as goat anti-mouse IgG-gold conjugate (6 nm) were used. **A.** Negative control. **B.** Disrupted cell. **C.** Repair mechanism via blebbing and shedding. Red arrows indicate membrane vesicles, green arrows indicate gold-labelled rHbl.

**Supplementary recordings**

Supplementary recordings 1-9 can be downloaded from https://doi.org/10.6084/m9.figshare.31537750.

# References

1. Tausch, F., Dietrich, R., Schauer, K., Janowski, R., Niessing, D., Märtlbauer, E., & Jessberger, N. (2017). Evidence for complex formation of the *Bacillus cereus* haemolysin BL components in solution. *Toxins (Basel)*, *9*(9). https://doi.org/10.3390/toxins9090288
2. Dietrich, R., Moravek, M., Bürk, C., Granum, P. E., & Märtlbauer, E. (2005). Production and characterization of antibodies against each of the three subunits of the *Bacillus cereus* nonhemolytic enterotoxin complex. *Appl Environ Microbiol*, *71*(12), 8214–8220. https://doi.org/10.1128/AEM.71.12.8214-8220.2005
3. Ge, S. X., Jung, D., & Yao, R. (2019). ShinyGO: a graphical gene-set enrichment tool for animals and plants. *Bioinformatics*, *36*(8), 2628–2629. https://doi.org/10.1093/bioinformatics/btz931
